# Structural and Biochemical Investigation of Class I Ribonucleotide Reductase from the Hyperthermophile *Aquifex aeolicus*

**DOI:** 10.1021/acs.biochem.1c00503

**Published:** 2021-12-23

**Authors:** Daniel Rehling, Emma Rose Scaletti, Inna Rozman Grinberg, Daniel Lundin, Margareta Sahlin, Anders Hofer, Britt-Marie Sjöberg, Pål Stenmark

**Affiliations:** † Department of Biochemistry and Biophysics, Stockholm University, S-106 91 Stockholm, Sweden; ‡ Department of Biochemistry and Biophysics, Umeå University, SE-907 36 Umeå, Sweden; § Department of Experimental Medical Science, Lund University, 221 00 Lund, Sweden

## Abstract

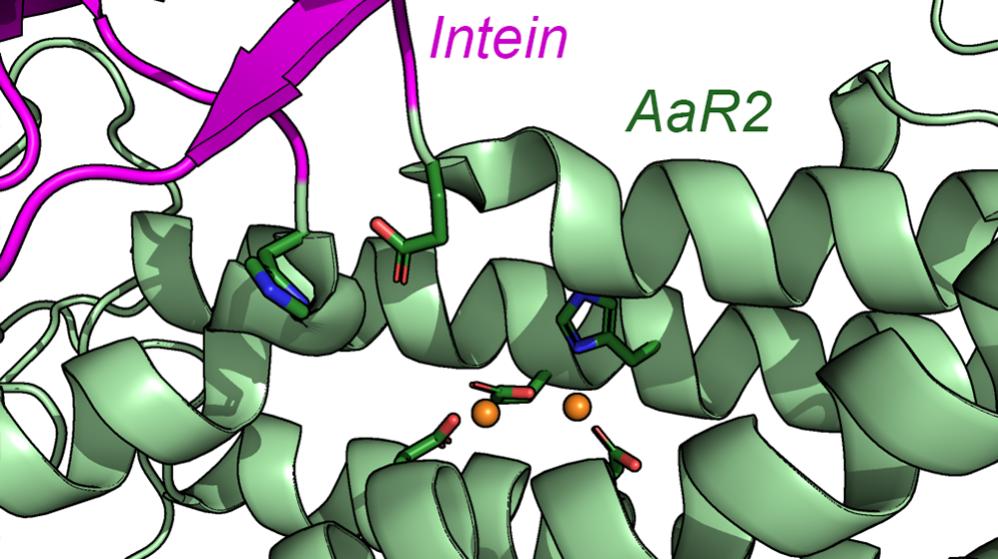

Ribonucleotide reductase (RNR) is an essential enzyme with a complex mechanism of allosteric regulation found in nearly all living organisms. Class I RNRs are composed of two proteins, a large α-subunit (R1) and a smaller β-subunit (R2) that exist as homodimers, that combine to form an active heterotetramer. *Aquifex aeolicus* is a hyperthermophilic bacterium with an unusual RNR encoding a 346-residue intein in the DNA sequence encoding its R2 subunit. We present the first structures of the *A. aeolicus* R1 and R2 (AaR1 and AaR2, respectively) proteins as well as the biophysical and biochemical characterization of active and inactive *A. aeolicus* RNR. While the active oligomeric state and activity regulation of *A. aeolicus* RNR are similar to those of other characterized RNRs, the X-ray crystal structures also reveal distinct features and adaptations. Specifically, AaR1 contains a β-hairpin hook structure at the dimer interface, which has an interesting π-stacking interaction absent in other members of the NrdAh subclass, and its ATP cone houses two ATP molecules. We determined structures of two AaR2 proteins: one purified from a construct lacking the intein (AaR2) and a second purified from a construct including the intein sequence (AaR2_genomic). These structures in the context of metal content analysis and activity data indicate that AaR2_genomic displays much higher iron occupancy and activity compared to AaR2, suggesting that the intein is important for facilitating complete iron incorporation, particularly in the Fe2 site of the mature R2 protein, which may be important for the survival of *A. aeolicus* in low-oxygen environments.

The enzyme ribonucleotide reductase (RNR) synthesizes the deoxyribonucleotides needed for DNA synthesis from their corresponding ribonucleotide precursors. RNR produces all four deoxyribonucleotides through a complex mechanism of allosteric regulation involving two distinct regulatory sites.^1,2^ Precise levels of dNTPs within cells are essential for cellular homeostasis and maintaining genomic integrity, with imbalances resulting in DNA replication errors, chromosomal abnormalities, and cell death.^3−5^ RNR harbors a free radical needed to catalyze the reduction of ribonucleotides to their corresponding deoxyribonucleotides, thereby providing the building blocks required for DNA synthesis and repair. To date, three classes of RNR (classes I–III) have been reported, which are distinguished from one another on the basis of how they generate and store the free radical needed to catalyze ribonucleotide reduction.^1,6,7^ The classes also differ from one another on the basis of which substrate they use. Class I RNRs exclusively use NDPs as the substrate; class III RNRs use NTPs directly, and in class II, there are some RNRs that use NDPs and others that use NTPs. Class I RNRs are composed of two proteins: a large “α-subunit” and a smaller “β-subunit”. The α- and β-subunits exist as homodimers, which are called R1 and R2, respectively, and combine to form a tetramer in the active state. Class I RNR synthesizes and provides a balanced supply of dNDPs through an intricate mechanism of allosteric regulation. The α-subunit contains three distinct ligand binding sites, which includes two allosteric sites in addition to the catalytic site.^8−10^ (1) The catalytic site (c-site) can bind four different substrates and is the site where ribonucleotide reduction occurs.^11^ (2) The substrate specificity site (s-site) is an allosteric site that binds nucleotide effectors (dGTP, dTTP, dATP, and ATP) and determines what substrate the c-site will reduce.^12,13^ (3) The activity site (a-site) is a second allosteric site, which regulates the overall activity of the enzyme by binding ATP to activate the enzyme or dATP that inhibits it.^8,14,15^ The actual reduction of ribonucleotides requires the generation of a radical in R2.^7^ Metal cofactors are involved in the generation of this radical, and in the case of class Ia RNRs, the R2 protein utilizes a diferric oxygen center (Fe–O–Fe).^16,17^ The radical is transferred over a large distance (30–35 Å) from Y• in R2 to a cysteine residue in the c-site of the R1 to form a thiyl radical needed to catalyze ribonucleotide reduction.^10,18^

Although class I RNR has been studied since the discovery of the enzyme in the early 1950s,^19^ many aspects of its function remain to be discovered. In fact, most of the attention has been focused on a few model RNRs from humans, other eukaryotes, and pathogens and there are therefore many types of RNRs that remain uncharacterized. Recently, we presented the structure of the R2 protein from *Clostridium botulinum*, the first example of the NrdBh evolutionary subclass,^20,21^ but the structure of the R1 protein, NrdAh, eluded us. In the same study, we presented low-resolution cryoEM evidence supporting the formation of inhibited complexes of the *C. botulinum* NrdAh/NrdBh of the same quaternary structure as the *Escherichia coli* NrdAg/NrdBg enzyme and a phylogeny supporting the relatively close evolutionary relationship between the *C. botulinum* NrdAh and the *E. coli* NrdAg enzymes.

In the study presented here, we structurally and biochemically characterized the class I RNR from *Aquifex aeolicus*, which is a member of the NrdAh evolutionary subclass. *A. aeolicus* is a hyperthermophilic bacterium that thrives in hot springs and underwater volcanoes, where temperatures often range between 85 and 95 °C.^22^*A. aeolicus* RNR is interesting from a structural and biochemical perspective because to tolerate such extreme conditions the enzyme must be remarkably stable. In addition, the DNA sequence of *A. aeolicus* R2 (AaR2) encodes a self-splicing intein, which after translation of the fusion protein is cleaved out via consecutive nucleophilic reactions resulting in the mature R2 protein.^23^ Inteins are largely considered to be selfish genetic elements and have been found in unicellular organisms from all domains of life.^24,25^ They are predominantly found in highly conserved regions of proteins that are essential for host survival (such as those involved in DNA replication, transcription, and maintenance), where they are less likely to be lost during evolution. The intein in AaR2 is found between two highly conserved residues (Glu228 and His231 in mature AaR2), which directly coordinate its catalytically essential di-iron center.

Here we present the first structures of *A. aeolicus* R1 (AaR1) and AaR2, in addition to biophysical and biochemical characterization of the *A. aeolicus* RNR enzyme. This is the first structure of an R1 protein from the NrdAh subclass and is also the first structure of a class Ia R1 from a thermophilic organism. Enzyme activity assays indicated that *A. aeolicus* RNR is maximally active at 79 °C, similar to the environmental temperature at which the organism lives. Utilizing GEMMA and size exclusion chromatography (SEC), we were also able to identify higher-order structures: the active α2β2 complex in the presence of ATP and a larger inactive α4β4 complex in the presence of dATP. While the active oligomeric state and activity regulation of *A. aeolicus* RNR are similar to those of other bacterial RNRs, the AaR1 structure has two interesting features: an a-site ATP cone that binds two ATP molecules rather than one, which a recent study of *E. coli* class Ia RNR released on bioRxiv suggests could be important for activity regulation, and a β-hairpin hook feature at the dimer interface, which can be seen as a defining feature of the NrdAh subclass. The AaR1 β-hairpin also displays a unique π-stacking interaction between the Tyr511 residues from both monomers, which are absent in other NrdAh sequences, and may be a factor that contributes to the high thermal stability of the protein. We expressed two AaR2 proteins: one in which the DNA sequence of the expressed protein lacked the intein (AaR2) and a second construct in which the nascent protein still included the intein sequence (AaR2_genomic). The purified proteins were of identical size, and their structures virtually identical, verifying that the intein is effectively removed following translation. Interestingly, the AaR2_genomic protein showed significantly higher iron occupancy and was twice as active as AaR2. It is likely that following translation, the di-iron site is loaded with iron, after which the intein cleaves itself out, generating mature AaR2. Importantly, this suggests that the native intein actually enhances iron incorporation in AaR2, particularly at the Fe2 site.

## Materials and Methods

### Cloning, Protein Overexpression, and Purification

DNA sequences encoding full length AaR1, AaR2, and AaR2_genomic were ordered from Genscript and cloned into a pET-28a(+) vector (Novagen) between *Nde*I and *Xho*I restriction sites. The resulting constructs contained an N-terminal His tag and thrombin cleavage site followed by the protein of interest.

AaR1, AaR2, and AaR2_genomic were expressed and purified using the same protocol. The proteins were expressed in *E. coli* BL21(DE3) R3 pRARE2 at 37 °C overnight, following induction by addition of 0.5 mM IPTG. The cultures were supplemented with 250 μM freshly prepared ammonium iron(II) sulfate hexahydrate 20 min prior to induction and again 2 h after induction. Cells were harvested and resuspended in lysis buffer [100 mM HEPES (pH 8.0), 500 mM NaCl, 10 mM imidazole, 10% glycerol, 0.5 mM TCEP, 25 units/mL benzonase, and 1 μL/mL Roche protease cocktail inhibitor], after which the cells were lysed by sonication. The lysate was clarified by centrifugation followed by filtration through a 0.45 μm membrane filter. The sample was loaded onto a 5 mL HisTrap HP column (GE Healthcare) equilibrated with running buffer [100 mM HEPES (pH 8.0), 500 mM NaCl, 10 mM imidazole, 10% glycerol, and 0.5 mM TCEP], and His-tagged protein was eluted with elution buffer [20 mM HEPES (pH 7.5), 500 mM NaCl, 500 mM imidazole, 10% glycerol, and 0.5 mM TCEP]. Fractions containing the protein of interest were pooled and loaded onto a Superdex 200 16/60 size-exclusion column (GE Healthcare) pre-equilibrated with 20 mM HEPES (pH 7.5), 300 mM NaCl, 10% glycerol, and 0.5 mM TCEP. Samples of pure protein were concentrated, after which TCEP was added to a final concentration of 2 mM. Protein aliquots were then flash-frozen in liquid nitrogen and stored at −80 °C. In addition, AaR2 and AaR2_genomic were expressed and purified again in exactly the same manner detailed above, with the exception that no reducing agent (TCEP) was added to any of the lysis, purification, or storage buffers.

### Crystallization and Data Collection

Aliquots of AaR1, AaR2, and AaR2_genomic [in 20 mM HEPES (pH 7.5), 300 mM NaCl, 10% glycerol, and 2 mM TCEP] were concentrated to 15 mg/mL. Aliquots of AaR2 and AaR2_genomic protein purified without a reducing agent [in 20 mM HEPES (pH 7.5), 300 mM NaCl, and 10% glycerol] were concentrated to 20 mg/mL. AaR1 was preincubated with 10 mM ATP and 10 mM magnesium chloride for 30 min at room temperature. All proteins were crystallized via hanging drop vapor diffusion (200 nL of protein and 200 nL of a crystallization solution) at 18 °C in 0.1 M sodium acetate (pH 4.5), 0.2 M lithium sulfate, and 30% PEG400 (AaR1) or in 0.1 M citrate (pH 4.0), 1.0 M lithium chloride, and 20% PEG6000 (all AaR2 and AaR2_genomic protein samples). Protein crystals were cryoprotected in mother liquor supplemented with 20% glycerol (all AaR2 and AaR2_genomic protein samples) or 20% ethylene glycol (AaR1) and flash-frozen in liquid nitrogen. Crystals of AaR1 appeared after 2 weeks, whereas all R2 crystals appeared after 2 days. For AaR1, AaR2, and AaR2_genomic crystals (protein purified with a reducing agent), X-ray diffraction data were collected at station I04 of the Diamond Light Source (Oxon, U.K.) equipped with a PILATUS-6M detector. The data sets were collected at 100 K on single crystals at a wavelength of 0.97 Å. For AaR2 and AaR2_genomic crystals (protein purified without a reducing agent), X-ray diffraction data were collected at station I03 of the Diamond Light Source equipped with an Eiger2 XE 16M detector. The data sets were collected at 100 K on single crystals at a wavelength of 0.97 Å.

### Structure Determination and Refinement

Data reduction and processing were carried out using DIALS^26^ and Aimless^27^ within the CCP4 suite.^28^ Structures were determined via molecular replacement with Phaser.^29^ The structure of *E. coli* ribonucleotide reductase R1 [Protein Data Bank (PDB) entry 1RLR] was used as the search model for the processed AaR1 data, and the structure of *E. coli* ribonucleotide reductase R2 (PDB entry 1AV8) was used as the search model for the processed AaR2 and AaR2_genomic data. Several rounds of model building and refinement were performed using Coot^30^ and Refmac5^31^ (AaR1 structure), during which waters and ligands were incorporated into the structures. The coordinates and structure factors for AaR1, AaR2_genomic, and AaR2 (protein purified with a reducing agent) have been deposited in the PDB as entries 7AGJ, 7AIK, and 7AIL, respectively. The coordinates and structure factors for AaR2_genomic and AaR2 (protein purified without a reducing agent) have been deposited in the PDB as entries 7Q39 and 7Q3C, respectively. Data processing and refinement statistics are listed in Table 1.

**Table 1:** Data Collection and Refinement Statistics

	AaR1	AaR2	AaR2_genomic	AaR2 without TCEP	AaR2_genomic without TCEP
PDB entry	7AGJ	7AIL	7AIK	7Q3C	7Q39
Data Collection^a^					
space group	*P*6_5_22	*P*4_3_2_1_2	*P*4_3_2_1_2	*P*4_3_2_1_2	*P*4_3_2_1_2
cell dimensions					
*a*, *b*, *c* (Å)	108.6, 108.6, 618.1	69.4, 69.4, 178.3	69.9, 69.9, 177.9	69.2, 69.2, 177.5	69.3, 69.3, 117.6
α, β, γ (deg)	90.0, 90.0, 120.0	90.0, 90.0, 90.0	90.0, 90.0, 90.0	90.0, 90.0, 90.0	90.0, 90.0, 90.0
resolution (Å)	94.0–2.70	69.4–1.73	65.0–2.10	48.9–2.15	47.2–2.10
no. of observations	1264409 (54406)	481951 (35817)	344127 (24924)	611398 (53455)	671542 (55886)
no. of unique observations	60783 (2944)	46576 (3385)	26837 (2102)	24392 (2058)	26173 (2084)
*R*_merge_	11.4 (99.1)	4.8 (137.8)	10.2 (75.0)	7.4 (251.9)	8.1 (294.2)
*R*_pim_	2.6 (23.4)	1.6 (46.6)	4.1 (31.6)	2.1 (70.3)	2.2 (81.0)
CC_1/2_	0.99 (0.84)	0.99 (0.81)	0.99 (0.96)	1.0 (0.69)	1.0 (0.67)
*I*/σ*I*	17.3 (1.4)	17.4 (1.6)	12.5 (2.3)	24.6 (1.4)	22.9 (1.2)
completeness (%)	99.6 (99.8)	100 (100)	99.7 (97.3)	100 (100)	100 (100)
redundancy	20.8 (18.5)	10.3 (10.6)	12.8 (11.9)	25.1 (26.0)	25.7 (26.8)
Refinement					
resolution (Å)	94.0–2.70	54.8–1.73	65.0–2.10	48.9–2.15	47.2–2.10
no. of reflections	57673	43386	25267	23110	24809
*R*_work_/*R*_free_ (%)	22.1/27.2	22.3/26.7	21.1/25.2	24.7/31.4	23.8/29.7
no. of atoms					
protein	11672	2792	2755	2676	2687
ligand	124	none	none	none	none
ions	2	1	2	2	2
water	78	193	112	84	69
*B*-factor (Å^2^)					
protein	71.5	42.2	53.8	66.8	67.4
ligands	90.2	n/a	n/a	n/a	n/a
ions	70.2	41.1	47.3	64.2	75.4
water	59.4	46.9	48.1	62.5	62.3
RMSD					
bond lengths (Å)	0.012	0.009	0.010	0.005	0.006
bond angles (deg)	1.21	1.53	1.56	1.20	1.35
Ramachandran (%)					
favored	97.1	99.1	94.7	96.2	91.2
allowed	2.7	0.9	5.3	3.8	8.5
disallowed	0.3	0	0	0	0.3

a Values in parentheses are for the highest-resolution shell.

### Size-Exclusion Chromatography

Fast protein liquid chromatography on a Superdex 200 PC 3.2/30 column (with a total volume of 2.4 mL) and ÄKTA prime system (GE Healthcare) was performed. The column was equilibrated with SEC buffer containing 20 mM Hepes (pH 7.4), 75 mM NaCl, 0.2 mM TCEP, and either 1 mM ATP and 5 mM MgCl_2_ or 0.5 mM dATP and 0.5 mM MgCl_2_. Samples (20 μL) containing AaR1, AaR2, or both subunits at equimolar concentrations in the presence of either 3 mM ATP and 10 mM MgCl_2_ or 2 mM dATP and 2 mM MgCl_2_ were preincubated for 5 min in 70 °C, centrifuged, and applied to the column at 7 °C with a flow rate of 0.1 mL/min. The same nucleotides that were added to the proteins were also included in the buffer to avoid dissociation of nucleotide-induced protein complexes during the run. Proteins were applied to the column at concentrations of 20–100 μM. Representative SEC chromatograms are shown in which AaR1, AaR2, and their equimolar mixtures at concentrations of 20 and/or 50 μM in the presence of ATP or dATP were used. The molecular weight was estimated on the basis of a calibration curve, derived from globular protein standards using high- and low-molecular weight SEC marker kits (Cytiva). Uncertainties are based on at least two SEC runs. To determine the complex stoichiometry, 0.05 mL fractions were collected during the AaR1 and AaR2 mixture runs. The top peak fraction was subjected to 12.5% sodium dodecyl sulfate–polyacrylamide gel electrophoresis (SDS–PAGE).

### GEMMA

Stock solutions of the R1 and R2 proteins were at concentrations of 24 and 42 mg/mL, respectively, which were high enough to use directly for GEMMA without prior desalting to ammonium acetate. Instead, they could be diluted directly into 100 mM ammonium acetate (pH 7.5) to working solutions of 1 mg/mL R1 protein and 0.5 mg/mL R2 protein. Nucleotide stock solutions were prepared by mixing equal concentrations of magnesium acetate and each nucleotide. The final mixtures used for GEMMA contained 0.25–1 μM R1 and/or R2 proteins (concentrations calculated per polypeptide), 100–400 μM Mg-nucleotide, 30 or 300 mM ammonium acetate (pH 7.5), and 0.005% Tween 20. The samples were preincubated for 2 min at 78 °C prior to GEMMA analysis. The GEMMA equipment consisted of a model 3480 electrospray aerosol generator, a model 3080 electrostatic classifier, a model 3085 nano-differential mobility analyzer, and a model 3025A ultrafine condensation particle counter using a sheath flow of 20 lpm and a sample flow of 1.5 lpm. An empirically determined protein particle density of 0.58 g/cm^3^ was used for diameter to mass conversion. The experiments were performed with a low differential pressure (1.7 psid) to reduce the flow rate and hence minimize the effect of nucleotides on the protein measurements. Each sample was scanned until a sufficient signal-to-noise ratio was obtained, and the raw data were plotted in GraphPad Prism 9.1.0 to be able to show several experiments in each panel.

### Electron Paramagnetic Resonance (EPR) and Ultraviolet–Visible (UV–vis) Spectroscopy

Measurements were performed with 214 μM AaR2 on a Bruker ELEXYS E500 spectrometer equipped with a coldfinger Dewar filled with liquid nitrogen (77 K), a modulation amplitude of 2 G, and a microwave power of 3.95 mW. The Xepr software package (Bruker) was used for data acquisition and processing of spectra. UV–vis spectra were recorded with 11 μM AaR2 at 25 °C on a PerkinElmer Lambda 35 spectrophotometer.

### Enzyme Activity Assays

Reaction conditions that give maximal activity were determined experimentally. For assays assessing the effect of temperature on the activity of *A. aeolicus* RNR, the reaction mixtures contained 2 μM AaR2, 4 μM AaR1, 50 mM Tris-HCl (pH 8, at room temperature), 100 mM KCl, 10 mM DTT, 10 mM magnesium acetate, and 3 mM ATP as an allosteric effector. Substrate CDP (0.8 mM) was added last to start the reaction. The reactions were run for 1–60 min (depending on the temperature) and stopped by the addition of methanol, and the mixtures transferred to ice. Reactions were run for 1 min at higher temperatures (≥75 °C), while reactions were run for either 2, 5, 10, 30, or 60 min at lower temperatures. Above 75 °C, the protein specific activity decreased with reaction time, indicating that the recombinant protein becomes destabilized at high temperatures.

For assays assessing the specificity regulation of *A. aeolicus* RNR, reaction mixtures contained 10 μM AaR2, 10 μM AaR1, 50 mM Tris-HCl (pH 6.8, at 79 °C), 100 mM KCl, 10 mM DTT, and 10 mM magnesium acetate. Four substrate assays were performed, in which the four substrates (CDP, ADP, GDP, and UDP) were simultaneously present in the assay mixture at concentrations of 0.5 mM each. The substrate mixture was added last to start the reactions. The indicated effectors (ATP, dATP, dTTP, dGTP, or a combination of two) were each present at a concentration of 2 mM. Reactions were run for 5 min at 79 °C and were stopped by heating the samples to 100 °C for 4 min. The conditions of these assays (protein concentrations and reaction time) were not optimal for obtaining maximal activity but allowed the detection and relative quantification of each of the four substrates.

Substrate conversion was analyzed by high-performance liquid chromatography (HPLC) using a Waters Symmetry C18 column (150 mm × 4.6 mm, 3.5 μm pore size) equilibrated with buffer A [10% methanol in 50 mM potassium phosphate buffer (pH 7.0) supplemented with 10 mM tributylammonium hydroxide]. Samples (15–50 μL) were injected and eluted at 0.4 mL/min and 10 °C with a linear gradient of buffer B [30% methanol in 50 mM potassium phosphate buffer (pH 7.0) supplemented with 10 mM tributylammonium hydroxide] from 0% to 30% over 40 min for the separation of CDP and dCDP or from 0% to 100% over 180 min for assays containing all four substrates. Compounds were identified by comparison with injected standards. Relative quantification was obtained by substrate and product peak height measurements in the chromatogram (UV absorbance at 254 or 271 nm). Specific activities of AaR2 were determined. Enzyme activity was calculated relative to the maximum activity observed for a given substrate.

For specific activity measurements comparing AaR2 and AaR2_genomic proteins (purified in the presence of TCEP) with AaR2 and AaR2_genomic proteins (purified without a reducing agent), the reaction mixtures contained the relevant AaR2 protein at 2 μM, 4 μM AaR1, 50 mM Tris-HCl (pH 8.0, at room temperature), 100 mM KCl, 10 mM DTT, 10 mM magnesium acetate, and 3 mM ATP as the allosteric effector and 0.5 mM CDP as the substrate. The reactions were run for 1 min at 79 °C, stopped by the addition of methanol, and then transferred to ice.

### Metal Quantification by TXRF

The metal content of AaR2 and AaR2 genomic proteins purified in the presence or absence of reducing agents was quantified using TXRF analysis on a Bruker PicoFox S2 instrument. For each solution, measurements on two independently prepared samples were performed. A gallium internal standard at 2 mg/L was added in an equal volume to the protein samples (final concentration of 200 μM) before the measurements. TXRF spectra were analyzed using the software provided with the spectrometer.

## Results

### Overall Structure of AaR1

**Figure 1:**
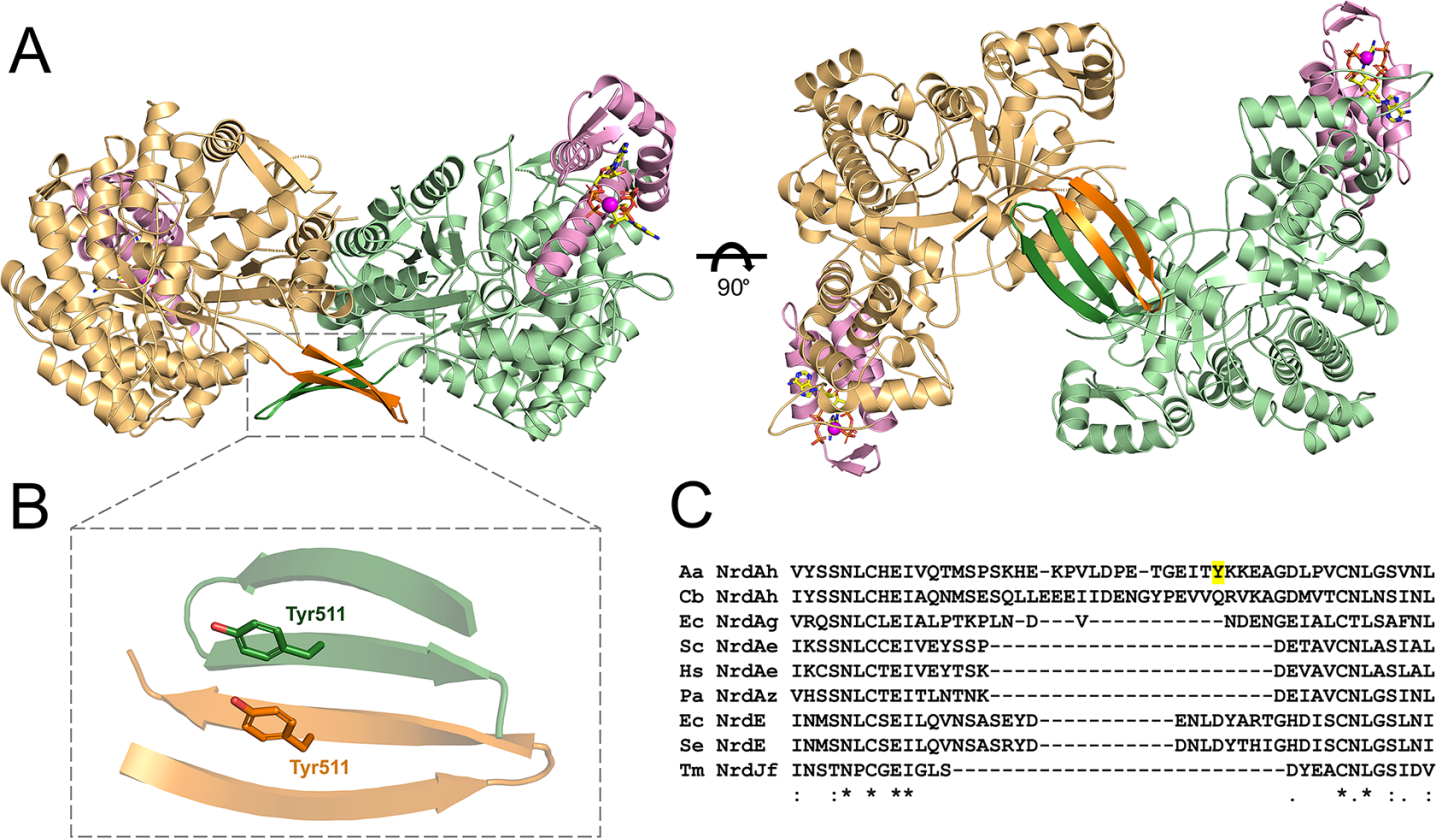
Crystal structure of AaR1 in complex with ATP. (A) AaR1 dimer with individual monomers shown as orange and green ribbons. An interesting β-hairpin “hook” structure at the dimer interface is colored dark orange and dark green (outlined by a dashed box). The ATP cone from each monomer is colored pink. The ATP ligands are depicted as stick models: yellow carbon atoms, red oxygen atoms, blue nitrogen atoms, and orange phosphorus atoms. Magnesium ions required for ligand coordination are displayed as magenta spheres. (B) Close-up of the AaR1 β-hairpin feature. Tyr511 residues from each monomer, which form a novel π-stacking interaction, are highlighted. (C) Sequence alignment of the AaR1 β-hairpin (Aa NrdAh) with the equivalent region from the sequences of *C. botulinum* NrdAh (Cb NrdAh), *E. coli* NrdAg (EcNrdAg), *Homo sapiens* NrdAe (Hs NrdAe), *P. aeruginosa* NrdAz (Pa NrdAz), *E. coli* NrdE (Ec NrdE), *S. enterica* NrdE (Se NrdE), and *T. maritima* NrdJf (Tm NrdJf). Tyr511 from AaR1 is highlighted in yellow.

The structure of AaR1 in complex with ATP was determined to 2.70 Å resolution. The statistics for data collection and refinement are listed in Table 1. The tertiary structure of AaR1 is a homodimer, where each monomer contains an “ATP cone” that houses two ATP molecules (Figure 1A). A structural similarity search was performed using the DALI web server.^32^ AaR1 displayed the highest *Z* scores with *E. coli*, human, yeast, and *Pseudomonas aeruginosa* R1, all of which have been extensively characterized, both structurally and biochemically.^9,15,18,33,34^ The individual AaR1 monomers interact, forming a dimer interface consisting of four α-helices and their associated loop regions, as well as a novel structural feature in which a β-hairpin (residues 495–515) from one monomer interacts with the equivalent β-hairpin structure from the opposing monomer, forming a four-stranded antiparallel β-sheet across the dimer interface (Figure 1B). Interestingly, this β-hairpin “hook” feature is not observed in structures of other class I enzymes from *E. coli*, *P. aeruginosa*, human, yeast, and *Salmonella enterica* or in the dimeric class II enzyme from *Thermotoga maritima* (Figure S1). The insert is, however, present in all NrdAh sequences but not in other NrdA sequences and can be seen as a defining feature of the NrdAh subclass (Figure 1C). Interestingly, closer structural analysis of the AaR1 β-hairpin revealed a unique π-stacking interaction between the Tyr511 of each monomer, a residue that is not present in other NrdAh sequences (Figure 1B,C).

During refinement, it was evident that there were areas in the structure where there was either very weak electron density or none at all, indicating a high degree of structural flexibility in those regions. These unmodeled areas are highly similar in both monomers. In monomer 1, they correspond to residues 275–289, 303–310, 332–340, 380–408, and 705–709, and in monomer 2, they correspond to residues 275–285, 303–310, 332–335, 380–408, and 707–709. One of these disordered regions corresponds to the additional large loop structure with a small 3_10_ helix present in the dimer interfaces of other R1 class Ia enzymes (residues 332–340). Two of these disordered regions are located in the proximity of the s-site (residues 274–289) and c-site (residues 303–310) of the protein. In addition, the C-terminal end of AaR1 (residues 788–799) is disordered in both monomers.

### AaR1 ATP Cone (a-site)

**Figure 2:**
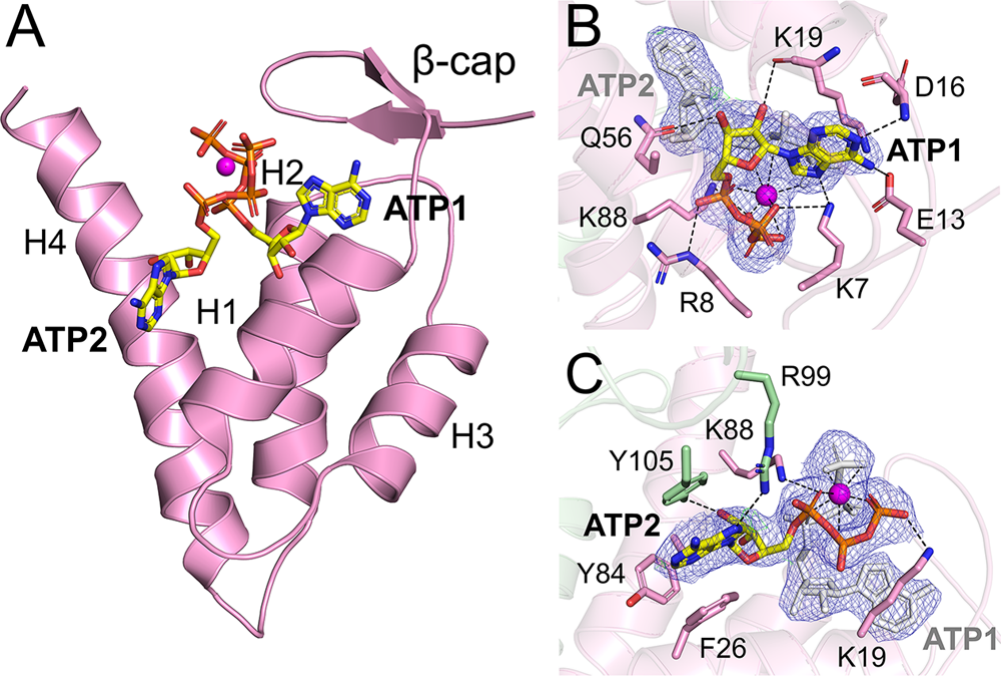
Crystal structure of the AaR1 ATP cone. (A) Crystallographic structure of the AaR1 ATP cone. The secondary structure elements, α-helices 1–4 (H1–H4, respectively) and two β-strands that form the “β-cap”, are highlighted. The two ATP molecules that bind in the ATP cone are shown as sticks: yellow carbon atoms, red oxygen atoms, blue nitrogen atoms, and orange phosphorus atoms. The magnesium ion that coordinates the phosphate groups is shown as a magenta sphere. Hydrogen bond networks of (B) ATP1 and (C) ATP2 within the ATP cone. The R1 monomer is shown as a green cartoon, with the ATP cone colored pink. Amino acids contributing to ligand coordination are depicted as sticks. Hydrogen bond interactions are shown as dashed lines. The 2*F*_o_ – *F*_c_ electron density map around the ATP molecules is contoured at 1.0σ (blue), and the *F*_o_ – *F*_c_ electron density maps are contoured at −3.0σ (red) and 3.0σ (green).

**Figure 3:**
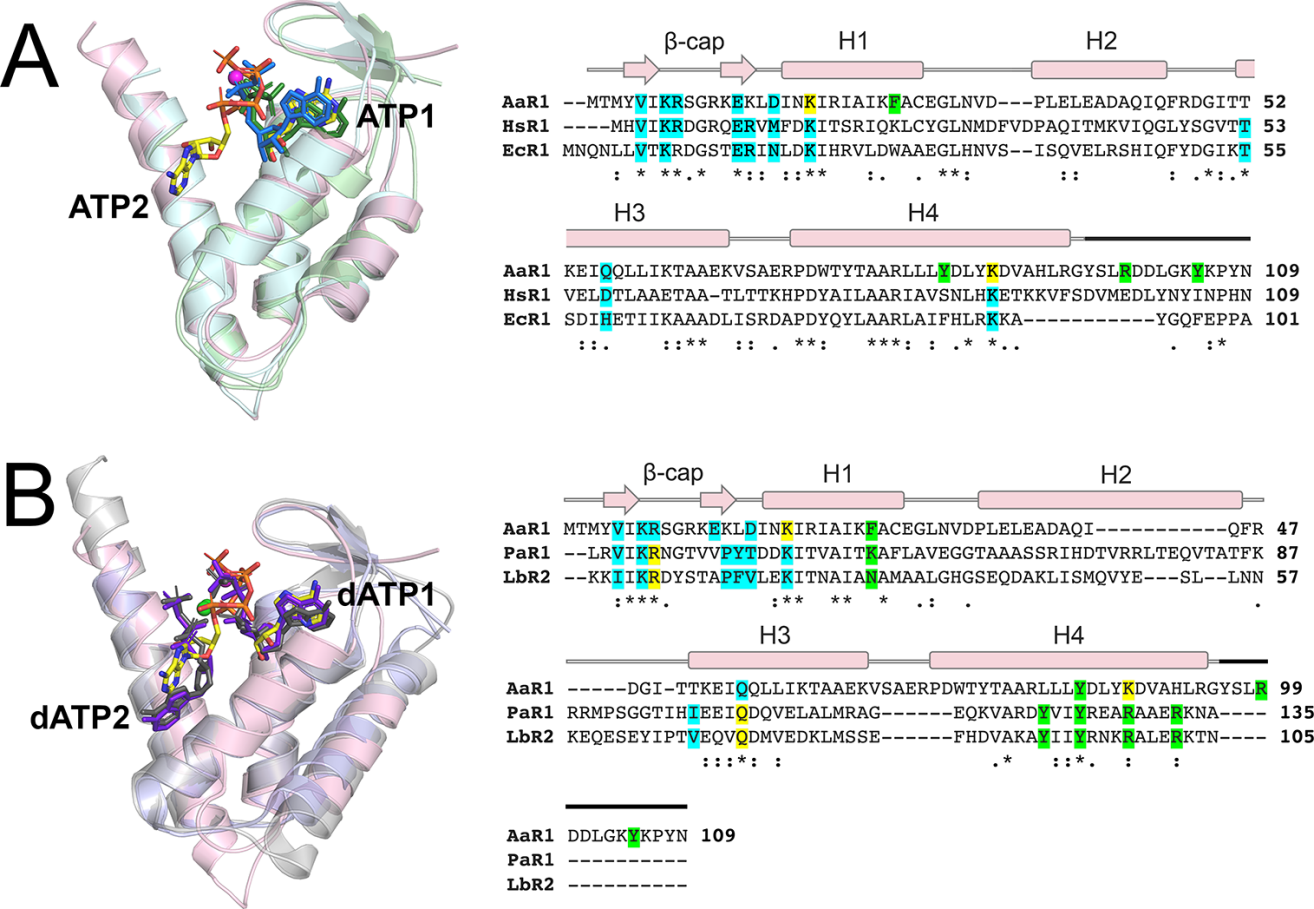
Comparison of the AaR1 ATP cone with those of structurally similar proteins. Superposition of the AaR1 ATP cone (light pink) with those of (A) human R1 (light blue, PDB entry 3HNE) and *E. coli* R1 (light green, PDB entry 3R1R) and (B) *P. aeruginosa* R1 (gray, PDB entry 5IM3) and *L. blandensis* R2 (purple, PDB entry 5OLK). The two ATP molecules from AaR1 are depicted as sticks: yellow carbon atoms, red oxygen atoms, blue nitrogen atoms, and orange phosphorus atoms. The single ATP ligands in human R1 and *E. coli* R1 are shown as blue and dark green stick models, respectively. The two dATP ligands present in the *P. aeruginosa* and *L. blandensis* structures are shown as dark gray and dark purple stick models, respectively. Magnesium ions are shown as pink (AaR1) or green spheres (*P. aeruginosa* and *L. blandensis*). Structure-based sequence alignments of the ATP cone region are presented. Blue highlighting indicates residues that interact with only ATP1, green highlighting those that interact with only ATP2, and yellow highlighting residues that interact with both ATP1 and ATP2. The secondary structure of the AaR1 ATP cone is shown above both sequence alignments. The dark black line indicates residues outside of the AaR1 ATP cone.

Each monomer in AaR1 contains an ATP cone (residues 1–95), which is composed of a four-α-helix bundle capped by a short β-hairpin (Figure 2A). The AaR1 ATP cone has electron density consistent with the presence of two ATP molecules, which is highly unusual, as R1 enzymes generally bind only a single ATP ligand in their ATP cones. However, a recent study released on bioRxiv presents an *E. coli* class Ia R1 structure with two ATP molecules bound in its ATP cone.^35^ Two bound dATP molecules have previously been observed in *P. aeruginosa* R1 (PaR1) and in the ATP cone in the *Leeuwenhoekiella blandensis* R2 structure (LbR2).^21,34^ In AaR1, the two ATP molecules (ATP1 and ATP2) in the ATP cone of AaR1 display very different hydrogen bond networks (Figure 2A). For ATP1, the side chains of Lys7 and Glu13 hydrogen bond with the N7 and N6 atoms of the adenine base, which is further supported by an interaction between its N1 atom and the main-chain nitrogen of Asp16. The ribose moiety of ATP1 is positioned between the side chain of Gln56 and the main-chain oxygen of Lys19. Finally, the triphosphate group of ATP1 is coordinated by interactions between Arg8 and the α-phosphate, Lys7 and the β-phosphate, and Lys88 and the γ-phosphate (Figure 2B). Comparisons between the AaR1 ATP cone and available structures from *E. coli*, humans, and *P. aeruginosa* and the ATP cone of *L. blandensis* R2 show that the core structure superimposes well (Figure 3A,B). In addition, the overall orientation of ATP1 in AaR1 is the same (with the exception of the phosphate groups) as the ATP in the human and *E. coli* R1 structures (Figure 3A).

The second ATP molecule (ATP2) binds the AaR1 ATP cone in a manner very different from that of ATP1. The adenine base is positioned by a π-stacking interaction between Phe26 and Tyr105, a CH/π interaction with Tyr84, and an interaction between the N7 position and the side chain of Arg99. Tyr105 also interacts with the ribose moiety of ATP2. Finally, the triphosphate group is positioned by interactions between Lys88 and the α-phosphate and Lys19 and the γ-phosphate (Figure 2C). In the AaR1 structure, residues Arg99 and Tyr105 are actually located outside of the ATP cone, on a loop (residues 96–109) connecting the last α-helix (H4) of the ATP cone to the catalytic domain of the R1 subunit (Figure 3A). In the ATP cone, there is also a single magnesium ion, which exhibits octahedral coordination with the phosphate groups of both ATP1 and ATP2 (Figure 2B,C). Human R1 lacks equivalent residues for Phe26 and Tyr105, which form an important π-stacking interaction with ATP2 in AaR1. *E. coli* R1, on the contrary, does have residues chemically similar to Phe26 (tryptophan in *E. coli* R1) and Tyr105 (phenylalanine in *E. coli* R1) (Figure 3A). This is in agreement with a study recently released on bioRxiv,^35^ which presents an *E. coli* class 1a R1 structure bound with two ATP molecules in the ATP cone. These structures are not yet publicly available; however, a hydrogen bond network for ATP2 in *E. coli* is presented, and the adenosine base is in fact coordinated by the same tryptophan and phenylalanine residues predicted in our sequence alignment (Figure 3A).^35^

Comparison of the AaR1 ATP cone with those from the PaR1 and LbR2 structures (each bound with two dATPs) shows that while ATP1 (AaR1) and dATP1 (PaR1 and LbR2) adopt the same orientation as what is observed for ATP in *E. coli* and human R1, the binding pose of ATP2 (AaR1) compared to that of dATP2 (PaR1 and LbR2) is significantly different (Figure 3B). As the phosphate groups of ATP2 and dATP2 point in opposite directions, the phosphate coordinating a magnesium ion also occupies a very different position in PaR1 and LbR2 compared to that in AaR1. In the PaR1 and LbR2 structures, dATP2 is positioned much deeper in the binding pocket. Analysis of the residues involved in ATP2 and dATP2 coordination indicates that while some of the residues required for ATP2 coordination are conserved the two important residues, Arg99 and Tyr105, have no equivalents at all in PaR1 or LbR2 structures (Figure 3B). Furthermore, there is a tyrosine residue that in PaR1 and LbR1 forms a CH/π interaction with the deoxyribose of dATP2, which is not conserved in AaR1 (Leu81) (Figure 3B). Overall, the structure of AaR1 is an important addition to the diversity of ATP cones that are being constantly discovered.

### Structural Flexibility in the c-Site and s-Site of AaR1

In R1 enzymes, the s-site of one monomer is in close proximity to the c-site of the second monomer, with important loop regions between them (Figure S2A). It is the binding of an effector to the s-site that dictates what substrate will be reduced in the c-site.^33^ There are two important loops (often referred to as loop 1 and loop 2), first identified in the structure of *E. coli* R1, that are critical for effector binding and cross-talk between the s-site and c-site^33,36,37^ (Figure S2B). In the AaR1 structure that lacks nucleotides in both the s-site and the c-site, loop 1 (residues 275–289) and loop 2 (residues 303–310) are disordered in both monomers (Figure S2A,B).

Comparison of the c-site of AaR1 with the R1 subunit of a recently determined *E. coli* RNR cryo-EM structure^18^ shows the overall secondary structure elements superimpose quite well (Figure S2B). The two tyrosines (Tyr730 and Tyr731, *E. coli* numbering) in the R1 subunit proposed to transfer the R2 radical to the c-site cysteine (Cys439, *E. coli* numbering) are conserved between the two structures. The two cysteines (Cys235 and Cys521, AaR1 numbering) that are known to become oxidized upon product formation in *E. coli* R1 form a disulfide bond in the AaR1 structure (Figure S2C). Analysis of the H-bond network for GDP in the *E. coli* R1 c-site shows that 75% of the residues involved in substrate coordination are conserved in AaR1 or have conserved physicochemical properties (Figure S2D). In the *E. coli* structure, three residues in loop 2 (which are disordered in AaR1) interact with GDP: the main-chain atoms of Gly299 and Ala301 and the side chain of Arg298. A sequence comparison indicates that Arg298 is conserved in AaR1, whereas Gly299 and Ala301 are lysine and serine, respectively, in the AaR1 structure. However, as it is the main-chain atoms of Gly299 and Ala301 that are involved in GDP coordination and their side chains actually point away from the substrate, the lysine and serine in AaR1 would still perform the same function.

Comparison of the s-site of AaR1 with *E. coli* R1 indicates that the majority of residues involved in effector recognition are conserved between the proteins (Figure S2E). The H-bond network for dTTP shows that it is positioned by residues from both R1 monomers. This includes residues from loop 1 from one monomer (Arg269 and Ile268, *E. coli* numbering) and loop 2 of the second monomer (Cys292*, *E. coli* numbering, where the asterisk signifies that the residue belongs to the second R1 monomer), all which are disordered in AaR1. The arginine and isoleucine residues are conserved in AaR1, whereas Cys292* is a valine in the AaR1 amino acid sequence. The only other differences in this region between *E. coli* R1 and AaR1 are Phe281 and Ser249* (*E. coli* numbering), which are Val291 and Ala261*, respectively, in AaR1 (Figure S2E). These differences are unlikely to significantly affect the substrate specificity as it is the main chains of Cys292* and Ser249* that interact with dTTP, and while valine is smaller than phenylalanine, both are still hydrophobic residues, meaning the overall function would likely be conserved.

### Radical Cofactor Site in AaR2

**Figure 4:**
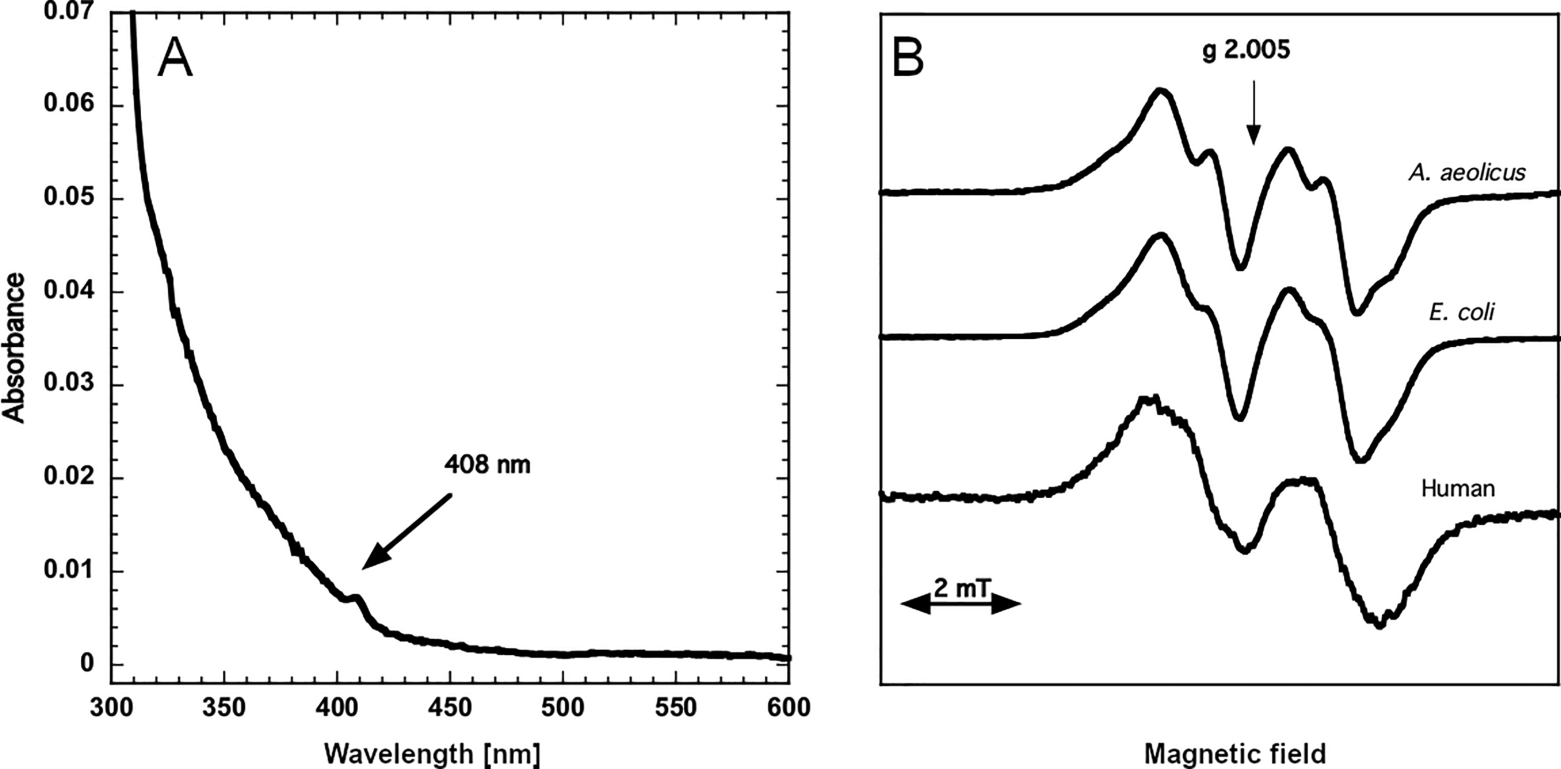
Spectral characterization of AaR2. (A) UV–vis spectrum of 11 μM AaR2 protein highlighting the tyrosyl radical absorption band at 408 nm. (B) EPR spectra at 77 K of 214 μM *A. aeolicus*, *E. coli*, and human R2 proteins. For comparison, all spectra were normalized to the same signal amplitude under nonsaturating conditions.

The AaR2 UV–vis spectrum shows a peak at 408 nm, and the EPR spectrum features an absorption peak major hyperfine coupling of 1.9–2.0 mT relating to a coupling to one of the β-protons in the tyrosyl residue and a smaller coupling of approximately 0.7 mT from the two 3,5-ring protons (Figure 4A,B). The AaR2 EPR spectrum is similar to the *E. coli* R2 spectrum (Figure 4B), but the magnetic interaction between the tyrosyl radical is stronger in AaR2, allowing the observation of an undisturbed signal at a higher power. The low tyrosyl radical content of AaR2 (0.05 Y•/AaR2) is likely the result of the low occupancy of iron in the active site.

### Temperature Dependence and Activity Regulation of *A. aeolicus* RNR Activity

**Figure 5:**
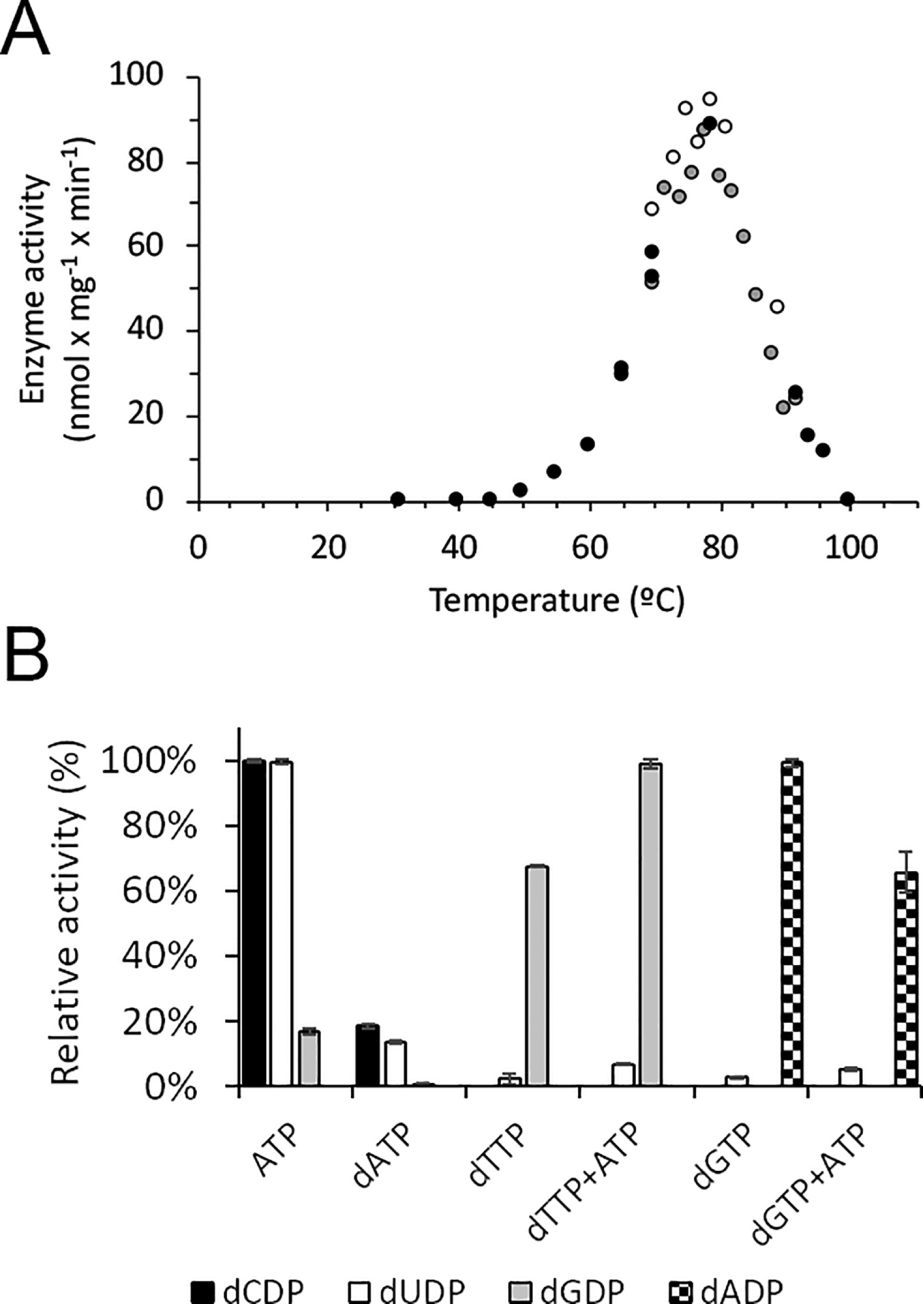
Temperature dependence and substrate specificity regulation of *A. aeolicus* RNR activity. (A) Temperature dependence. CDP reduction was assayed at different temperatures in assay mixtures containing 2 μM AaR2, 4 μM AaR1, 0.8 mM CDP, and 3 mM ATP as the allosteric effector. Data sets from three independent experiments are plotted (black, white, and gray circles, each color representing a different data set). (B) Specificity regulation. The maximum specific activities for the conversion of CDP, UDP, GDP, and ADP to their corresponding dNDPs were 57, 23, 45, and 39 nmol mg^–1^ min^–1^, respectively. Error bars indicate the standard deviation of three independent experiments. The maximum specific activity with CDP in panels A and B is not identical, because the reaction conditions were different. The assay mixtures contained 10 μM AaR2, 10 μM AaR1, four substrates (CDP, ADP, GDP, and UDP) at concentrations of 0.5 mM each, and the indicated allosteric effector (ATP, dATP, dTTP, dGTP, or a combination of the two) at 2 mM. See Materials and Methods for details.

To investigate the temperature dependence of *A. aeolicus* RNR, enzyme activity was tested in the presence of 3 mM ATP and 0.8 mM CDP at temperatures ranging between 30 and 100 °C. The enzyme was inactive at temperatures below 50 °C. The highest specific activity was obtained at 79 °C (Figure 5A), which is very close to the growth temperature of *A. aeolicus* (85–95 °C). The relatively low specific activity of approximately 100 nmol min^–1^ (mg of protein)^−1^ is a consequence of the small amount of tyrosyl radical (0.05 Y•/AaR2) and corresponds to a *k*_cat_ of approximately 2 s^–1^ per Y•. To determine the substrate specificity regulation of *A. aeolicus* RNR, we performed assays in which the four substrates (CDP, ADP, GDP, and UDP) were simultaneously present in the assay mixture at concentrations of 0.5 mM each and the effectors of interest (ATP, dATP, dTTP, dGTP, or a combination of two) were present at concentrations of 2 mM each. The results clearly demonstrate that the regulation of the s-site is quite similar to that of most other RNRs,^2,6^ where ATP induces the reduction of CDP and UDP while dTTP and dGTP induce the reduction of GDP and ADP, respectively (Figure 5B). dATP at 2 mM clearly inhibits activity, presumably through binding to the a-site. We performed a series of activity assays with CDP as the substrate, in which we titrated ATP and dATP into the reaction mixtures, and established the roles of ATP as a positive and dATP as a negative allosteric regulator of *A. aeolicus* RNR (Figure S3A,B). The addition of ATP and dTTP increases the level of GDP reduction, whereas the level of ADP reduction decreases in the presence of ATP and dGTP, likely due to the formation of inhibitory dADP (Figure S3C).

### Comparison of the AaR2 Structure with the Structure Generated from a Construct in Which the Nascent Protein Still Contains the Intein

The DNA sequence of the *A. aeolicus* R2 subunit encodes a 346-residue self-splicing intein, nearly as large as the mature AaR2 protein itself. Interestingly, prior to its removal, the intein separates two highly conserved residues (Glu228 and His231, in mature AaR2 numbering), which directly coordinate the catalytically essential di-iron center in mature AaR2 (Figure S4). We expressed, purified, and determined the X-ray crystal structures of two AaR2 proteins: one in which the DNA sequence of the expressed protein lacked the intein (AaR2) and a second in which the nascent protein still included the intein sequence (AaR2_genomic). Autocatalytic splicing of the intein in AaR2_genomic generates a peptide bond between Leu229 and Cys230, which thereby brings Glu228 and His231 (Figure S4) into position to serve as ligands for the dinuclear metal site. The crystal structure of AaR2 provides clear insights into the overall structure and the architecture of its di-iron center, whereas the AaR2_genomic construct enabled us to study the splicing efficiency of the intein in a non-native host and whether its removal has any effect on the overall structure and di-iron site of the mature protein.

**Figure 6:**
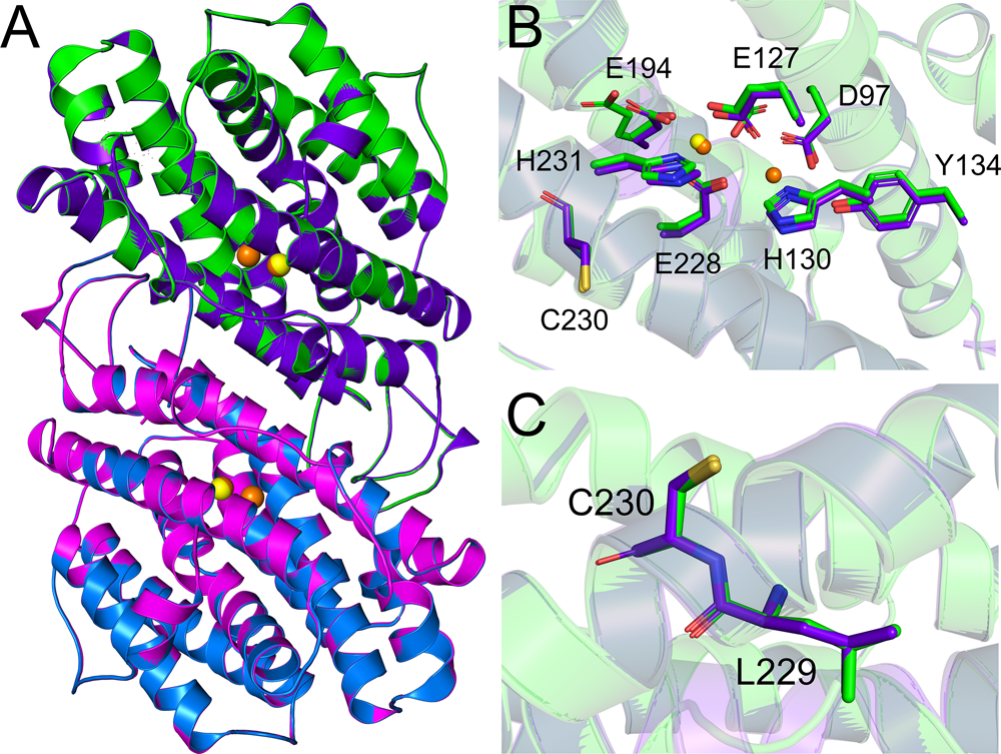
Comparison of mature AaR2 and AaR2_genomic crystal structures. (A) Superposition of AaR2 (individual monomers colored green and blue) with AaR2_genomic (individual monomers colored purple and magenta). The iron ions of AaR2 are shown as yellow spheres, and those of AaR2_genomic are colored orange. (B) Comparison of iron coordination in AaR2 (green) and AaR2_genomic (purple). Amino acids contributing to metal coordination are depicted as sticks: red oxygen atoms, dark blue nitrogen atoms, and gold sulfur atoms. (C) Comparison highlighting the peptide bond formed between Leu229 and Cys230 following intein removal.

Following purification, SDS–PAGE analysis of AaR2 and AaR2_genomic proteins showed that they were of the same size (Figure S5), indicating that for the AaR2_genomic construct, the intein had successfully spliced itself out of the nascent protein sequence to generate mature AaR2. We crystallized the mature AaR2 and AaR2_genomic proteins and determined the X-ray crystal structures to resolutions of 1.73 and 2.10 Å, respectively. Both structures have missing electron density at their N-terminus (residues 1–7) and C-terminus (residues 328–350). The statistics for data collection and refinement are listed in Table 1. The tertiary structure of both variants is a homodimer, consistent with what has been observed for related R2 proteins.^38−40^ A structural similarity search was performed using the DALI web server,^32^ which indicated that AaR2 and AaR2_genomic are most similar to R2 proteins from *E. coli*, humans, mice, *Bacillus halodurans*, and *Chlamydia trachomatis*. Superposition of AaR2 with AaR2_genomic shows the overall structures are virtually identical, with a low RMSD value of 0.15 Å (Figure 6A). Analysis of the AaR2 di-iron site showed clear electron density for only one iron atom (termed Fe1), which was built in with an occupancy of 0.9 (Figure 6B). Fe1 is coordinated by three carboxylates (Glu127, Glu194, and Glu228), a histidine residue (His231), and one ordered water molecule. Interestingly, residues Glu127 and Glu194 display alternate conformations (Figure S6A). In contrast, the di-iron site of the AaR2_genomic structure showed electron density consistent with the presence of two iron atoms, termed Fe1 and Fe2 (Figure 6B). The two irons are coordinated by four carboxylates (Asp97, Glu127, Glu194, and Glu228) and two histidines (His130 and His231) (Figure S6B). A higher occupancy is observed for Fe1 compared to that of Fe2. Fe1 was modeled with an occupancy of 0.8 and is positioned by residues Glu127, Glu194, Glu228, and His231, in addition to an ordered water molecule, whereas Fe2 was modeled with an occupancy of 0.25 and is coordinated by Asp97, Glu127, His130, and Glu228. The distance between the two iron atoms is 3.8 Å. The phenolic oxygen of the presumed radical harboring tyrosine (Tyr134) is positioned 3.1 Å from Asp97 and is 4.7 Å from the nearest iron atom (Figure S6B). In the case of both AaR2 and AaR2_genomic, the electron density for the metal sites is somewhat less defined than the core of the protein, indicative of the partial occupancy and/or X-ray photoreduction. Comparison of the AaR2 di-iron site with that of AaR2_genomic shows that the iron-coordinating residues occupy identical positions, with the exception of residues Glu194 and Glu127, which as noted above display alternate conformations in the AaR2 structure. Fe1 occupies the same position in both structures (Figure 6A). Iron-coordinating residues Glu228 and His231 and residues Leu299 and Cys230 (which in the nascent protein sequence is located at either end of the intein) occupy the same positions in both AaR2 and AaR2_genomic (Figure 6B,C).

### Increasing Iron Incorporation in AaR2 Proteins

**Figure 7:**
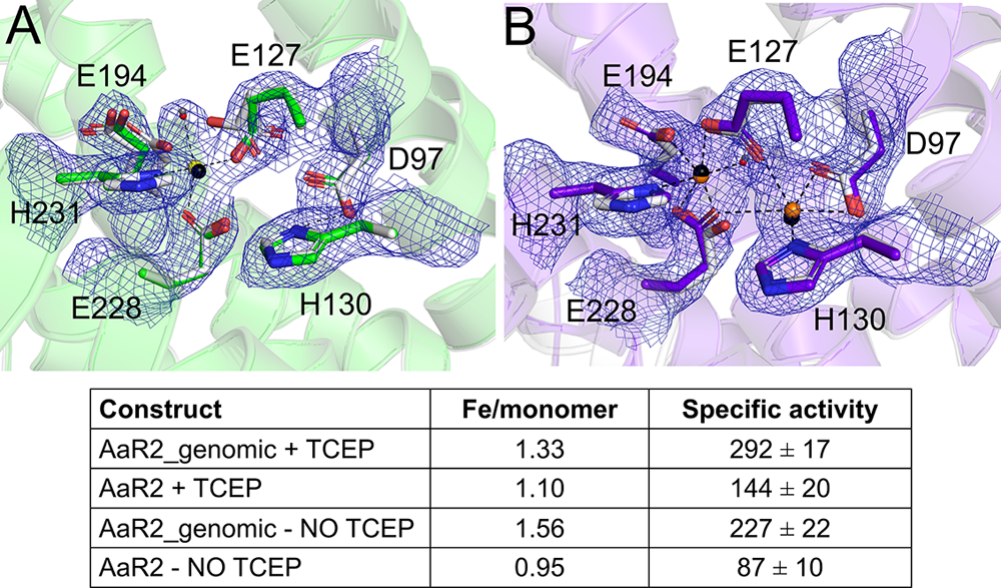
Comparison of iron incorporation in AaR2 and AaR2_genomic proteins in the presence or absence of a reducing agent. (A) Comparison of AaR2 proteins purified with (white) or without (green) TCEP. (B) Comparison of AaR2_genomic proteins purified in the presence (white) or absence (purple) of TCEP. The hydrogen bond interactions (dashed lines) of iron ions with the active site and the electron density maps correspond to the proteins purified without a reducing agent. Relevant water molecules in each structure are shown as red spheres (proteins purified without TCEP) or dark gray (proteins purified with TCEP). Iron atoms are colored yellow (AaR2_no TCEP), orange (AaR2_genomic no TCEP), or black (AaR2 and AaR2_genomic with TCEP). Hydrogen bond interactions are displayed as dashed lines. The 2*F*_o_ – *F*_c_ electron density maps are contoured at 1.0σ, and the *F*_o_ – *F*_c_ electron density maps are contoured at 3.5σ. A table shows the levels of Fe incorporation per monomer [determined by TXRF (see Materials and Methods)] for each protein. The specific activities (nanomoles per milligram per minute) of the different AaR2 and AaR2_genomic samples (using 0.5 mM CDP as a substrate at 79 °C) are also shown (see Materials and Methods).

The low radical content and activity of AaR2 is likely the result of the absent or poor occupancy observed for the second iron in the active site in our AaR2 and AaR2_genomic structures. As *A. aeolicus* is a hyperthermophile, the incorporation of iron during expression in *E. coli* is likely not optimal. To increase the metal and radical content of the AaR2 protein, we tried heating the sample in the presence of Fe^2+^ and also attempted unfolding and refolding the protein in the presence of Fe^2+^. Unfortunately, heating the sample proved to be ineffective, and the attempts to emulate the natural folding conditions of the protein were not successful. Another possibility for the low radical content and poor occupancy for the second iron is that the presence of a reductant (TCEP) during purification may have partially reduced the tyrosyl radical and/or iron cluster. To address this, we purified AaR2 and AaR2_genomic again in the complete absence of reducing agent. We crystallized the new protein samples using the same crystallization condition used for the AaR2 and AaR2_genomic proteins purified in the presence of TCEP. We determined the X-ray crystal structures of TCEP free AaR2 and AaR2_genomic to resolutions of 2.15 and 2.10 Å, respectively. Both structures have missing electron density at their N-terminus (residues 1–7) and C-terminus (residues 328–350). The statistics for data collection and refinement are listed in Table 1. Superposition of AaR2 with AaR2_genomic (without TCEP) with their corresponding structures (with TCEP) shows the overall structures are virtually identical, with low RMSD values of 0.22 and 0.25 Å for the AaR2 and AaR2_genomic proteins, respectively. Analysis of the AaR2 (no TCEP) di-iron site showed clear electron density for only one iron atom (Fe1), which was built in with an occupancy of 1.0 (Figure 7A), similar to what was observed in the previous structure (Figure S6B). The AaR2_genomic structure (no TCEP) on the contrary showed clear electron density for both Fe1 and Fe2 in the active site (Figure 7B). This is a significant improvement from the AaR2_genomic (with TCEP) structure, where the density for Fe2 was much weaker [modeled in at 0.25 occupancy (Figure S6B)]. To complement these results, we also performed TXRF analysis to determine the Fe content of the samples and also determined the specific activity of all four protein samples with the preferred substate CDP. The specific activity and Fe content of the AaR2 and AaR2_genomic proteins were similar with or without TCEP, indicating that their proportions of active radical are likely also comparable. However, the TXRF analysis clearly indicates that the AaR2_genomic proteins consistently had higher Fe contents and the activity was double that of the AaR2 proteins, regardless of whether the proteins were purified with a reducing agent (Figure 7).

### Oligomerization of *A. aeolicus* RNR

**Figure 8:**
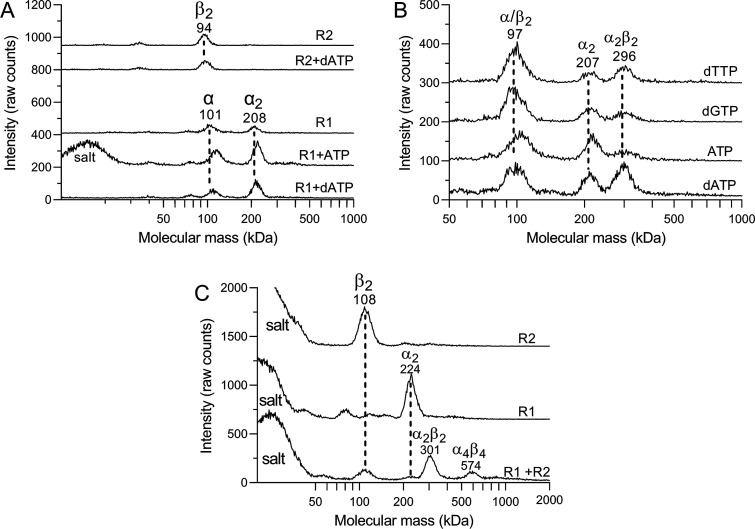
Oligomerization of *A. aeolicus* RNR by GEMMA. (A) Allosteric effector-induced oligomerization of individual R1 and R2 proteins measured at a concentration of 0.25 μM (theoretical molecular masses of the R1 and R2 polypeptides are 93 and 42 kDa, respectively). The experiments were performed in the absence or presence of 300 μM ATP or 100 μM dATP. (B) Oligomerization of R1–R2 interaction with and withoutthe indicated allosteric effectors at 100 μM for dNTPs or 300 μM for ATP. (C) Analysis of 1 μM R1 and/or R2 in the presence of 400 μM dATP. The proteins were analyzed both individually and in combination. The indicated molecular masses are in kilodaltons and with suggested oligomerization complexes indicated on the top of each peak. When a peak consists of two nonresolved species, we have written the dominating one. Experiments A and B were performed in the presence of 300 mM ammonium acetate (pH 7.5), whereas the ammonium acetate concentration was decreased to 30 mM in experiment C. Magnesium acetate was present at concentrations equimolar to those of the nucleotides. The traces are vertically distributed to be able to plot several experiments in each panel. Theoretical molecular masses of α_2_, β_2_, α_2_β_2_, and α_4_β_4_ complexes are 190, 90, 280, and 560 kDa, respectively.

Gas-phase electrophoretic mobility macromolecule analysis (GEMMA) studies of *A. aeolicus* RNR at room temperature showed that AaR2 is a dimer and AaR1 is in a monomer–dimer equilibrium (Figure 8A). The samples contained 300 mM ammonium acetate, which helped to stabilize the proteins, and were incubated at 78 °C prior to being analyzed to resolve protein aggregates formed at lower temperatures. Allosteric effectors stimulated R1 dimerization, although complete conversion to dimers was not obtained under the testing conditions (Figure 8A). In contrast, AaR2 was in a dimeric form irrespective of the presence of an effector (Figure 8A). ATP was used at a higher concentration (300 μM) than dATP (100 μM) because it generally has a lower affinity than dNTPs for binding to R1 proteins.^2^ A limitation with GEMMA is that nonvolatile components such as nucleotides form clusters when the protein solution is sprayed out in the gas phase and the liquid evaporates from the fine droplets formed in the process. This restricts how much nucleotides can be used in the experiment, and in the experiment with 300 μM ATP, a salt peak formed from clusters of ATP and cations was observed. These clusters can also form around proteins during evaporation and are responsible for the small shift of the protein peaks to higher masses as observed in the ATP experiment. From R1–R2 interaction studies, we observed the formation of an α_2_β_2_ heterotetramer complex (Figure 8B). However, the formation of the α_2_β_2_ complex was inefficient, and no complexes larger than that were observed. To force the complex formation, we increased the protein and dATP concentrations 4-fold and decreased the ammonium acetate concentration from 300 to 30 mM (Figure 8C). The conversion to the α_2_β_2_ form was nearly complete under these conditions, and some larger complexes also appeared. The formation of the larger complexes required both subunits and did not occur with R1 or R2 alone. The molecular mass was 574 kDa, which is approximately double that of the α_2_β_2_ complex, indicating that it has an α_4_β_4_ quaternary structure.

**Figure 9:**
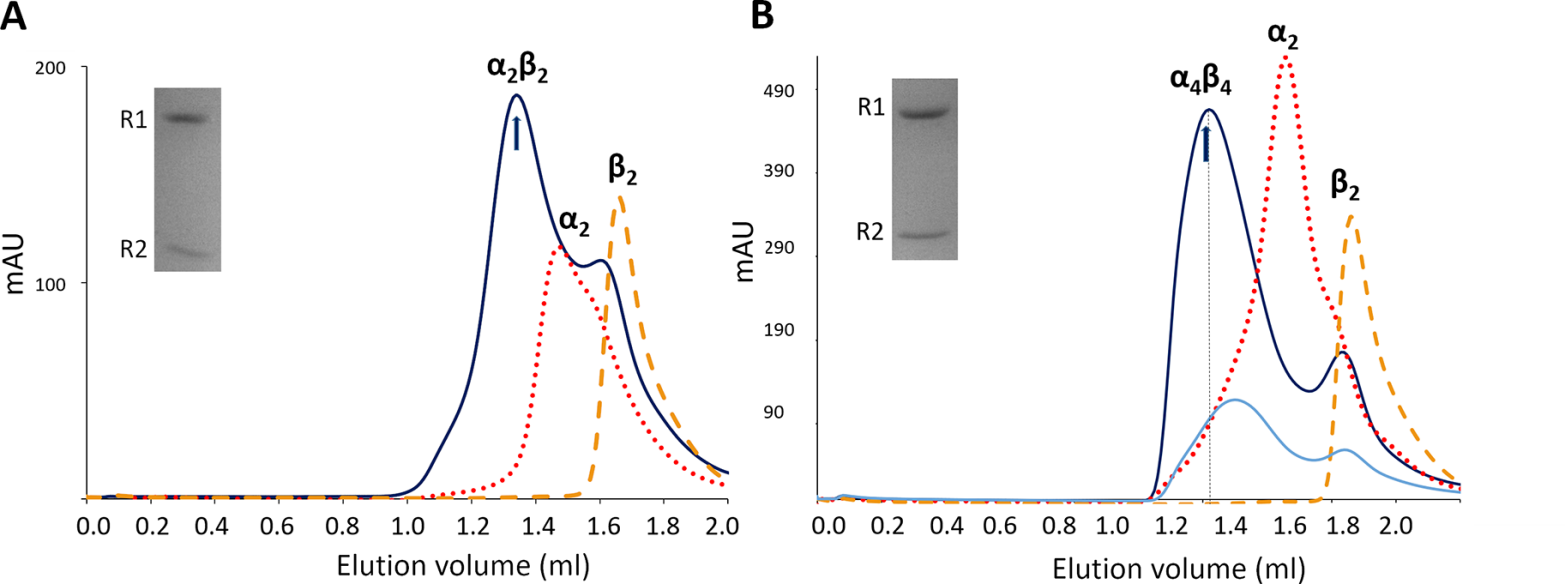
Size-exclusion chromatography of AaR1 and AaR2 proteins in the presence of allosteric effectors ATP and dATP. (A) AaR1 (red dotted), AaR2 (orange dashed), or an equimolar combination of AaR1 and AaR2 (blue solid) was preincubated separately or mixed together at a concentration of 20 μM in the presence of 5 mM ATP. (B) Protein concentrations of 20 μM (light blue) and 50 μM (dark blue) were assayed in the presence of 1 mM dATP. Samples were incubated at 70 °C for 5 min, centrifuged, and applied to the column equilibrated with SEC buffer at 7 °C. The closest estimated complex stoichiometries are shown above the peaks. Representative traces are shown. The insets show SDS–PAGE gels of the eluted protein complexes. Elution positions of fractions applied to the gel are indicated by arrows.

To complement the GEMMA analyses of oligomer formation and to allow measurements at higher protein and nucleotide concentrations, we performed analytical SEC (Figure 9). The SEC experiments confirmed the GEMMA results. In the presence of both ATP and dATP, the AaR1 is in a dimer–monomer equilibrium, with the predominant species being a dimer (184 ± 5 kDa). The AaR2 subunit eluted as a dimer (70 ± 0.5 kDa). When the AaR1 and AaR2 proteins were mixed at an equimolar concentration of 20 μM, they formed a larger complex with a calculated molecular weight of 360 ± 177 kDa in the presence of ATP. To verify its composition and stoichiometry, we analyzed peak fractions eluted from SEC via SDS–PAGE. Both AaR1 and AaR2 were visible on the gel in a 1:1 molar ratio (Figure 9, inset). On the basis of its molecular weight and subunit composition, the ATP-induced complex is conceivably an α_2_β_2_ structure (Figure 9A). When dATP was the allosteric effector, the formed complex was larger with a calculated molecular mass of 428 ± 10 kDa. However, the peak was broad, indicating a mixture of high-molecular weight oligomers. Increasing the concentrations of R1 and R2 to 50 μM each resulted in a 590 ± 291 kDa complex, which did not increase in size when the protein concentrations were further increased to 100 μM (Figure S7). SDS–PAGE of the peak fraction confirmed a 1:1 AaR1:AaR2 molar ratio in the complex, strengthening the conclusion from the GEMMA experiment that this is an α_4_β_4_ complex (Figure 9B).

## Discussion

In this study, we determined the first structures of *A. aeolicus* proteins AaR1 and AaR2 and characterized the *A. aeolicus* RNR. *A. aeolicus* RNR is highly interesting from a structural and biochemical perspective as to tolerate the extreme temperatures at which *A. aeolicus* lives, the enzyme needs to be extremely stable. Furthermore, the DNA sequence of the R2 protein encodes a self-splicing intein, which after translation is cleaved out via consecutive nucleophilic reactions, resulting in mature AaR2. This structural and biochemical analysis provides insights into how thermophile class I RNRs work and answers the question of whether the intein in the nascent R2 protein affects the final structure of the protein. The AaR1 structure also provides the first structural information about an R1 protein from the NrdAh subclass.

While the active oligomeric state and activity regulation of *A. aeolicus* RNR are similar to those of other bacterial RNRs, the AaR1 structure has two interesting features: a β-hairpin hook structure at the dimer interface and an a-site ATP cone housing two ATP molecules instead of one. When compared with related R1 proteins, we noted that AaR1 had an additional structural element at the dimer interface consisting of two intertwining β-hairpin structures (or β-hairpin “hook”), which is not present in the other structures. Sequence analysis of this feature revealed that the β-hairpin is present in all NrdAh sequences but not in other NrdA sequences and is thus a defining feature of the NrdAh phylogenetic subclass. However, closer structural inspection of the AaR1 β-hairpin also revealed a π-stacking interaction between the Tyr511 from each monomer, which is unique to the AaR1 sequence, and may be a factor contributing to the high thermal stability of the protein.

Interestingly, analysis of the AaR1 ATP cone indicated clear electron density for the presence of two ATP molecules, which is unusual as R1 enzymes usually only bind to a single ATP.

As noted previously, two dATP molecules have been observed in PaR1 and in the ATP cone of LbR2.^21,34^ Moreover, the LbR2 ATP cone was able to bind two ATP molecules, as shown using isothermal titration calorimetry. Detailed analysis of the AaR1 ATP cone showed that the position of ATP1 adopts the same orientation as ATP1 from human and *E. coli* R1.^9,33^ This was also the case for dATP1 from PaR1 and LbR2. Overall, this observation was expected as the residues responsible for ATP1 binding are almost entirely conserved between these structures. Interestingly, a recent study released on bioRxiv^35^ suggests that *E. coli* class Ia R1 is also capable of binding two ATP molecules in the ATP cone. The hydrogen bond network for ATP2 in *E. coli* R1 is shown to involve π-stacking interactions with Phe87, Phe97, and Trp28 (*E. coli* numbering), as well as hydrogen bond interactions with Arg10, Arg24, Lys9, and Lys21 (*E. coli* numbering). All of these interactions were predicted in our sequence alignment comparing the AaR1 and *E. coli* ATP cones, with the exception of Arg24, which is Ile22 in AaR1 (Figure 3A). In the recent *E. coli* R1 study, it was proposed that the binding of ATP2 is critical for the ability of ATP to dismantle the inactive α_4_β_4_ complex. Specifically, the binding of ATP2 to the second site (site 2) in the ATP cone results in numerous conformational changes that break the α–β interface, which frees β, restoring activity. In contrast, binding of dATP creates a pocket for β on α, which traps β_2_ in the inactive α_4_β_4_ state. Furthermore, mutations of residues in site 2 that are critical for positioning the adenosine base of ATP2 (W28A, F28A, and F97A) were shown to disrupt activity regulation.^35^ In light of this study and the structural similarities in ATP2 binding in AaR1 and EcR1, this strengthens the argument that the binding of ATP2 in the AaR1 ATP cone is legitimate and that it is not merely an artifact of crystallization.

Comparison of ATP2 with dATP2 from PaR1 and LbR2, as well as further structural and sequence comparison with human R1, indicates that ATP2 coordination in AaR1 differs from that of the other enzymes. It is evident that human R1 lacks equivalent residues needed for the important π-stacking interaction with ATP2 observed in AaR1 and *E. coli* R1.^35^ PaR1 and LbR2 have only one aromatic residue capable of π-stacking at this position rather than two, which is observed in AaR1 and *E. coli*. There are also no residues at the same position in the PaR1 and LbR2 structures. Overall, this analysis offers an explanation for why the ATP cone of human R1 is capable of coordinating only a single ATP molecule and why the binding of ATP2 in AaR1 differs significantly from the binding of the second nucleotide in PaRa and LbR2. It is evident that all R1 ATP cones share a primary site for ATP binding and that for those capable of binding two ATP molecules, the position of the secondary site can differ.

In the AaR1 structure, there were no bound effector ligands in the s-site of AaR1, in spite of ATP being present during crystallization, which should be capable of binding at the s-site. There was also nothing bound in the c-site, which is unsurprising as the protein was crystallized without substrates. We also noted two areas of disordered density located near the s-site and c-site, which in other R1 structures are referred to as loop 1 and loop 2. In the apo form of *T. maritima* R1, loop 2 is in a partially closed conformation that does not leave enough room for substrate binding.^41^ However, when an effector binds, loop 2 shifts toward the s-site and is capable of adopting three different conformations depending of which allosteric effector is bound, creating enough room for the substrate to bind in the c-site.^42^ Following substrate binding, loop 2 shifts again, back toward the c-site.^13,42^ As there are no ligands bound in the c-site or s-site of AaR1, it is therefore not entirely surprising that loop 1 and loop 2 are disordered in the AaR1 structure. Superposition of AaR1 with the R1 subunit of the *E. coli* RNR cryo-EM structure^18^ showed the two tyrosines in the R1 subunit proposed to transfer the radical to the c-site cysteine are conserved. Interestingly, the two cysteine residues that are known to become oxidized upon product formation are oxidized in the AaR1 structure, as evidenced by the disulfide bond between Cys235 and Cys521.

Comparison of residues comprising the AaR1 c-site with those of *E. coli* R1 showed that the majority of residues involved in substrate coordination are conserved in AaR1 or have conserved chemical properties.^18^ Comparison of the s-site of AaR1 with *E. coli* R1 indicated that the majority of residues involved in effector recognition are conserved between the proteins.^18^ The high degree of structural similarity between the s- and c-sites of *E. coli* and *A. aeolicus* R1 makes sense in the context of our activity assays performed in the presence of four substrates and different allosteric effectors, which demonstrate that *A. aeolicus* RNR substrate specificity is in line with the canonical s-site regulation,^2^ which is highly conserved throughout RNRs of all three classes and is an ancient feature that appeared early on in RNR evolution.^6^

Our activity assays indicated that *A. aeolicus* RNR is maximally active at 79 °C, and at this temperature, the enzyme was clearly inhibited by dATP and active in the presence of ATP. Our GEMMA and SEC data showed that the active oligomeric state of *A. aeolicus* RNR is an α_2_β_2_ heterotetramer, similar to other class I RNRs. In bacteria, RNR is an active heterotetramer when ATP is bound to the a-site. When dATP binds to the a-site, RNRs form higher-order oligomers, resulting in enzyme inhibition.^14^ The inactive RNR complexes vary greatly and may include only R1, only R2, or both R1 and R2.^20^ Our results suggest that the inactive *A. aeolicus* RNR oligomeric state in the presence of dATP is α_4_β_4_ and thus belongs to the category depending on both subunits for the formation of an inhibited oligomer. The inactive RNR structure from *C. botulinum*,^20^ which belongs to the NrdAh/NrdBh subclass, and the inactive RNR structure from *E. coli*,^43^ which belongs to the phylogenetically close NrdAg/NrdBg subclass, show that they have ring-shaped structures.

Apart from residues needed for the autocatalytic splicing mechanism, a major part of the intein is a homing endonuclease. In the case of the AaR2 intein, it is of the LAGLIDADG type. Homing endonucleases are mobile genetic elements frequently found in inteins and as free-standing genes in self-splicing group I introns that transmit their genes horizontally within a host population.^44^ The inteins and group I introns found in R2 genes are primarily located in the vicinity of the two regions that harbor metal-ligating residues and the tyrosyl radical residue, in AaR2 corresponding to residues Glu127, His130, Tyr134, Glu228, and His231 (Figure S4). The inteins in phage R2 proteins (indicated in Figure S4) are all of the LAGLIDADG type, whereas the homing endonucleases in the group I introns (indicated in Figure S3) are primarily of the HNH and GIY-YIG types.

Analysis of the AaR2 and AaR2_genomic structures showed they were virtually identical, indicating that the presence of the intein in the native protein (and its removal) does not ultimately affect the overall structure of the enzyme. However, it was evident that the presence of the intein in the construct seemed to influence the occupancy of the iron, particularly the Fe2 site, which had 25% occupancy in the AaR2_genomic structure but was empty in AaR2. It was evident from those structures that we were not able to fully occupy the di-iron site in AaR2, which is likely the reason that only 5% of the protein contained active radical according to EPR analysis. The host organism *A. aeolicus* lives at high temperatures in geothermal vents, so it is likely that the incorporation of iron during expression in *E. coli* at 37 °C is poor. We tried heating the protein in the presence of Fe^2+^ and unfolding and refolding the protein in the presence of Fe^2+^ to emulate the natural folding conditions of AaR2, which proved to be unsuccessful. As the presence of reducing agents in our purification buffers might have affected iron incorporation, we purified AaR2 and AaR2_genomic again in the absence of reducing agent. The resulting crystal structures showed AaR2_genomic had full occupancy for both Fe1 and Fe2, indicating the presence of TCEP in the purification buffer was in fact affecting Fe2 binding during crystallization. AaR2 on the contrary had no density for the Fe2 site as was the case with the previous structure. TXRF analysis showed that AaR2_genomic consistently had a higher Fe content, and the activity was double that of AaR2, regardless of whether or not the proteins were purified with a reducing agent. This clearly shows that the intein in AaR2_genomic enhances the incorporation of iron into the mature protein, particularly in the Fe2 site. It is therefore most likely that following translation, the di-iron site is loaded with iron, after which the intein cleaves itself out, generating mature AaR2. It may be that the presence of the intein between residues in the di-iron site makes it more open and/or accessible, allowing for more effective iron incorporation. This may be advantageous for *A. aeolius* under certain conditions. In particular, the bacterium is capable of surviving in environments in which the oxygen concentration is as low as 7.5 ppm.^45^ Clearly, a fully loaded di-iron site in such a situation would be extremely important, as this in combination with oxygen is necessary for the formation of the active tyrosyl radical required for RNR activity.

In summary, we present the first structures of AaR1 and AaR2 proteins and characterization of the *A. aeolicus* RNR heterotetramer. *A. aeolicus* RNR is maximally active at 79 °C, consistent with the environmental conditions of its host organism. Using GEMMA and SEC, we were able to identify higher-order structures: in the presence of ATP, the detected complex was most consistent with the expected size of the active α_2_β_2_ complex, and in the presence of dATP, a much larger complex was detected, closer to the expected size of the inactive α_4_β_4_ complex. While the active oligomeric state and activity regulation of *A. aeolicus* RNR are similar to those of other bacterial RNRs, the AaR1 structure also revealed two interesting structural features: an ATP cone that houses two ATP molecules and a β-hairpin hook feature at the dimer interface with a unique π-stacking interaction not observed in other members of the NrdAh subclass. The structure of AaR1 is an important inclusion to the diversity of R1 structures and ATP cones, which have continuously been discovered in recent years. Importantly, the structures of AaR2 and AaR2_genomic proteins in addition to determination of their metal content and specific activity, indicates that AaR2_genomic has significantly higher iron content and activity compared to AaR2. This suggests that the presence of the intein in the nascent protein sequence enhances incorporation of iron into the mature AaR2 protein, particularly in the Fe2 site, which may be important for the survival of *A. aeolicus* in low-oxygen environments.

## Supporting Information Available

Comparison of the AaR1 structure with those of related R1 proteins from *E. coli*, humans, yeast, *S. enterica*, and *T. maritima*; Comparison of the s-site and c-site of AaR1 with *E. coli* R1; AaRNR activity assays showing the effect of the allosteric effectors ATP and dATP; AaR2 protein sequence highlighting the position of an intein that is present in the nascent protein; SDS–PAGE analysis of AaR2 and AaR2_genomic proteins following purification; hydrogen bond network with electron density for the di-iron sites of AaR2 and AaR2_genomic; and SEC analysis of AaR1 and AaR2 using different protein concentrations (PDF)

## Accession Codes

The protein structures presented herein have been deposited in the Protein Data Bank as entries 7AGJ, 7AIK, 7AIL, 7Q3C, and 7Q39.

## Abbreviations

RNR: ribonucleotide reductase
ATP: adenosine triphosphate
s-site: specificity site
a-site: activity site
c-site: catalytic site
AaR1: *A. aeolicus* R1
AaR2: *A. aeolicus* R2
GEMMA: gas-phase electrophoretic mobility macromolecular analysis
TCEP: tris(2-carboxyethyl)phosphine
PDB: Protein Data Bank
RMSD: root-mean-square deviation.
