# Supplementary material for: Structural and Biochemical Investigation of Class I Ribonucleotide Reductase from the Hyperthermophile Aquifex aeolicus
Source: Biochemistry. 2021 Dec 23;61(2):92–106. doi: 10.1021/acs.biochem.1c00503 (PMC8772380; doi:10.1021/acs.biochem.1c00503)
Supplement: Supplementary file 1 — bi1c00503_si_001.pdf [file bi1c00503_si_001.pdf]

Structural and biochemical investigation of class I ribonucleotide reductase from the hyperthermophile *Aquifex aeolicus*

Daniel Rehling[1*], Emma Rose Scaletti[1*], Inna Rozman Grinberg[1*], Daniel Lundin[1], Margareta Sahlin[1], Anders Hofer[2], Britt-Marie Sjöberg[1] and Pål Stenmark[1,3]

[1]Department of Biochemistry and Biophysics, Stockholm University, S-106 91, Stockholm, Sweden.

[2]Department of Biochemistry and Biophysics, Umeå University, SE-907 36 Umeå, Sweden.

[3]Department of Experimental Medical Science, Lund University, Lund 221 00, Sweden.

Correspondence should be addressed to Britt-Marie Sjöberg, email: britt-marie.sjoberg@dbb.su.se* or Pål Stenmark, e-mail: stenmark@dbb.su.se*

* These authors contributed equally to this work

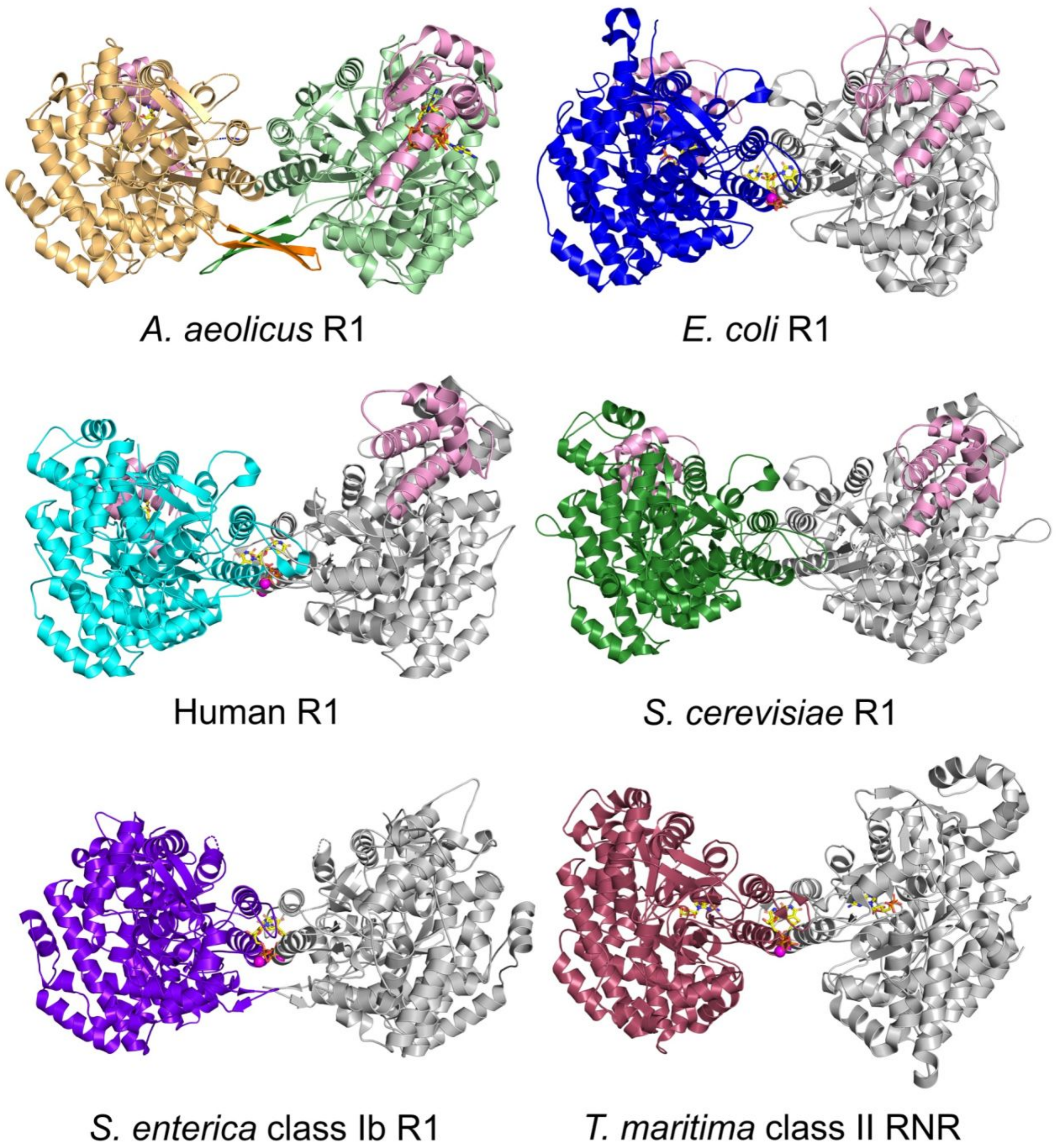

Figure S1. Comparison of AaR1 with structurally similar R1 proteins. Individual monomers are depicted as ribbon representations, coloured orange and green (AaR1), blue and grey (*E. coli* R1*,* PDB ID: 6W4X), cyan and grey (human R1*,* PDB ID: 3HNF), green and grey (*S. cerevisiae* R1*,* PDB ID: 3PAW), purple and grey (*S. enterica* class Ib R1, PDB ID: 1PEQ) and burgundy and grey (*T. maritima* class II RNR, PDB ID: 1XJE). The ATP-cones of AaR1, *E. coli* R1, human R1 and *S. cerevisiae* R1 are shown in pink. The ATP ligands are depicted as stick models; carbon atoms yellow, oxygen atoms red, nitrogen atoms blue and phosphorous atoms orange. Magnesium ions required for ligand coordination are displayed as magenta spheres. Nucleotide ligands in each structure are shown as sticks; carbon atoms yellow, oxygen atoms red, nitrogen atoms blue and phosphorus atoms orange. *A. aeolicus* R1 is bound with ATP (ATP-cone, a-site), *E. coli* R1 is bound with GDP (c-site) and dTTP (s-site), human R1 is bound with dTTP (s-site) and dATP (a-site), *S. enterica* class Ib R1 is bound with dTTP (s-site) and *T. maritima* class II RNR is bound with GDP (c-site) and dTTP (s-site).

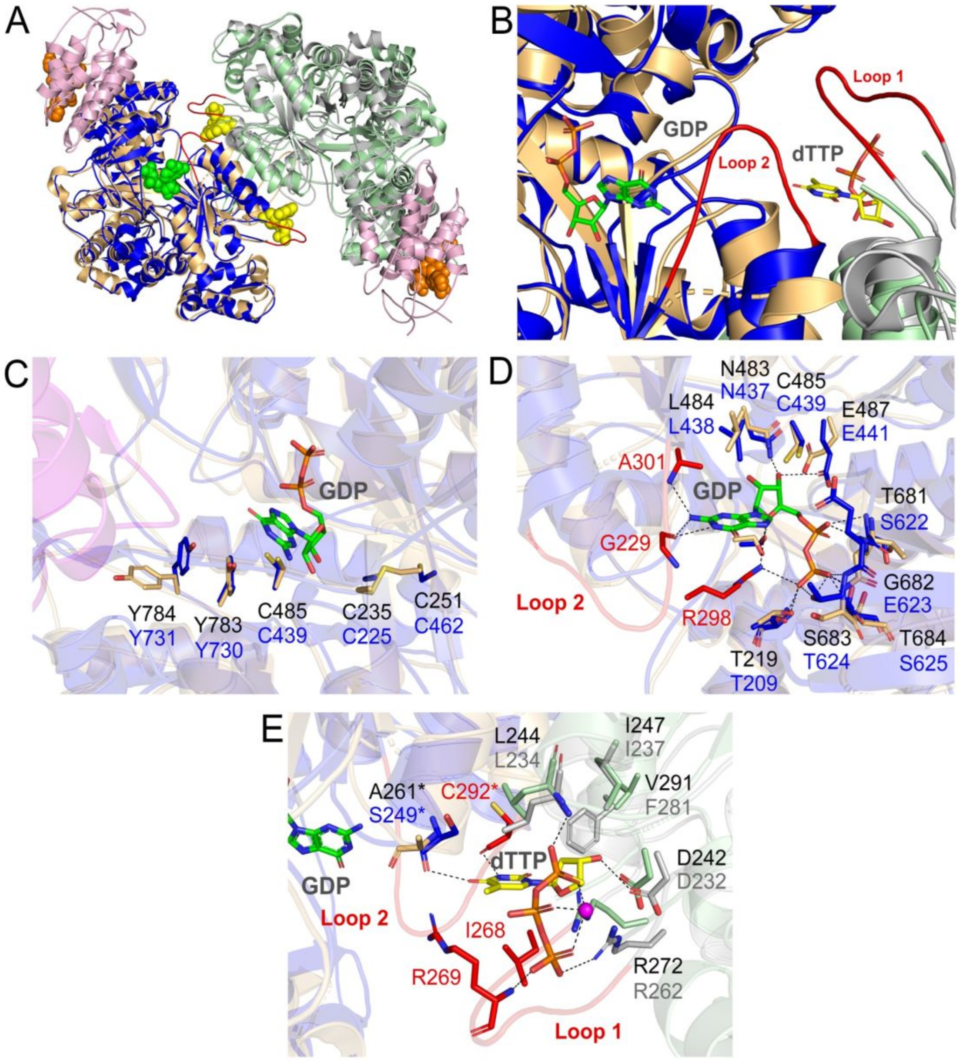

Figure S2. Comparison of AaR1 with *E. coli* R1 highlighting the c-site and s-site. (A) Superposition on AaR1 and *E. coli* R1, where Individual monomers are depicted as ribbon representations, coloured orange and green (*A. aeolicus*) and blue and grey (*E. coli,* PDB ID: 6W4X). The ATP-cones (a-site) from AaR1 and *E. coli* R1 are coloured pink. ATP in the AaR1 a-site is shown in dark orange, GDP in the *E. coli* c-site is shown in green and dTTP in the *E. coli* s-site is shown in yellow. *E. coli* R1 loop 1 and loop 2, which are involved in the c-and s-sites, are coloured red. These loops are disordered in the AaR1 structure. (B) Close up view of *E. coli* R1 loop 1 and loop 2, showing their positions relative to the c-site (GDP) and s-site (dTTP). Nucleotide ligands in this panel are shown as sticks; carbon atoms yellow (dTTP) or green (GDP), oxygen atoms red, nitrogen atoms blue and phosphorus atoms orange. (C) Comparison between AaR1 and *E. coli* R1 highlighting the two radical transferring tyrosines, the actual cysteine involved in ribonucleotide reduction (Cys485, AaR1 numbering) and two cysteines (Cys235 and Cys521, AaR1 numbering) that are oxidized upon product formation. The R2 subunit of *E. coli* RNR structure (PDB ID: 6W4X) is shown as a magenta cartoon. (D) Coordination of GDP in the *E. coli* R1 c-site compared the equivalent residues from AaR1. Hydrogen bond interactions are shown as dashed lines. (E) The hydrogen bond network of dTTP in the *E. coli* R1 s-site compared with the equivalent residues from AaR1. Residues from monomer 2 in both structures are labelled with an asterisk (*).

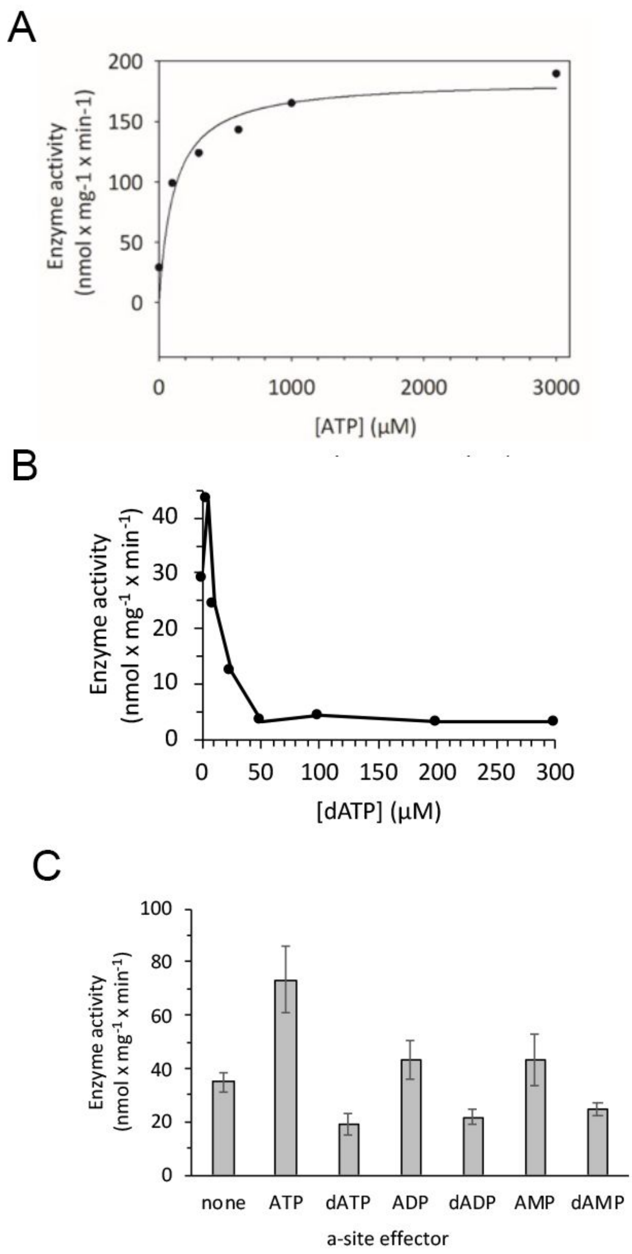

Figure S3. Activity of *Aquifex aeolicus* RNR in the presence of allosteric effectors. (A) ATP activates the enzyme with a K_L of 113 µM. From a direct plot of activity versus concentration of effector, the K_L value for binding of ATP to the a-site, was calculated in SigmaPlot using the equation: V=Vmax x [dNTP]/(K_L + [dNTP]). (B) dATP activated the enzyme when used at a low concentration (5 µM), presumably by binding to the s-site and was inhibitory at 10 µM concentration and higher. The reaction mixtures in A and B contained 4 µM AaR1, 4 µM AaR2 genomic, 0.5 mM CDP substrate and the indicated amount of allosteric effector ATP or dATP. The reaction was run for 1 minute at 79 °C. **(C)** GDP reduction in presence of s-site saturating dTTP effector (2 mM), and 2 mM of the indicated adenosine nucleotides. ATP activated the enzyme, dATP, dADP and dAMP were inhibitory and ADP and AMP didn't affect the activity. Since dTTP saturated the s-site, the activation and inhibition of RNR activity occured via the a-site. Assay mixtures contained 4 µM AaR1, 2 µM AaR2 and 0.8 mM GDP substrate. The reaction was run for 1 minute at 79 °C. Error bars show the standard deviation of three experiments.

```
        10         20         30         40         50         60         70
MEKTEKNELV RKLIFNPQGD REASKRKIIK GNPTNIFELN EIKYSWAFDL YKLMGFTNFW IPEEIQMLED
        80         90        100        110        120        130        140
RKQYETVLSD YEKRAYELVL SFLIALDSFQ VDMLKEFGRM ITAPEVEMAI TAQEFQESVH AYSYQFILES
       150        160        170        180        190                   210
VVDPVKADEI YNYWREDERL LERNKVIAEL YNEFIRKPNE ENFIKATIGN YILESLYFYS GFAFFYTLGR
       220        229
QGKMRNTVQQ IKYINRDELC FIEGTEVLTK RGFVDFRELR EDDLVAQYDI ETGEISWTKP YAYVERDYEG

SMYRLKHPKS NWEVVATEGH EFIVRNLKTG KERKEPIEKV KLHPYSAIPV AGRYTGEVEE YDLWELVSGK

GITLKTRSAV KNKLTPIEKL LIVLQADGTI DSKRNGKFTG FQQLKFFFSK YRKINEFEKI LNECAPYGIK

WKKYERQDGI AYTVYYPNDL PIKPTKFFDE WVRLDEITEE WIREFVEELV KWDGHIPKDR NKKKVYYYST

KEKRNKDFVQ ALCALGGMRT VVSRERNPKA KNPVYRIWIY LEDDYINTQT MVKEEFYYKG KVYCVSVPKG
                230        244        254        264        274        284
NIVVRYKDSV CIAGNCHVTL FRNIINTLRK ENPELFTPEI EKWIVEYFKY AVNEEIKWGQ YVTQNQILGI
       294        304        314        324        334        344    350
NDVLIERYIK YLGNLRITQI GFDPIYPEVT ENPLKWIDEF RKINNTKTDF FQAKPQTYSK ANELKW
```

Figure S4. The nascent protein sequence of AaR2_genomic showing the position of the intein within AaR2. Residues responsible for coordinating iron are shaded *blue*. The tyrosyl radical residue is shaded *green*. The intein which is post-translationally cleaved out of the AaR2 protein is shaded *grey*. Two cysteine residues participating in this process are coloured *red*. Insertion sites self-splicing elements in other R2 sequences are indicated with arrowheads (intein, *red*, and group I intron, *black*); intein insertion sites are from several Pectobacterium phages, Corynebacterium phage Darwin, Pseudanabaena phage and Tenacibaculum phage, and group I intron insertion sites are from *Bacillus amyloliquefaciens, Bacillus cereus* spp, *Bacillus glycinifermentans, Bacillus licheniformis, Bacillus methylotrophicus, Bacillus subtilis* spp, *Bacillus thuringiensis* spp, *Streptococcus pneumoniae* spp, *Thiovulum* sp, Acinetobacter phage, Brochothrix phage, and Enterobacteriophage T4.

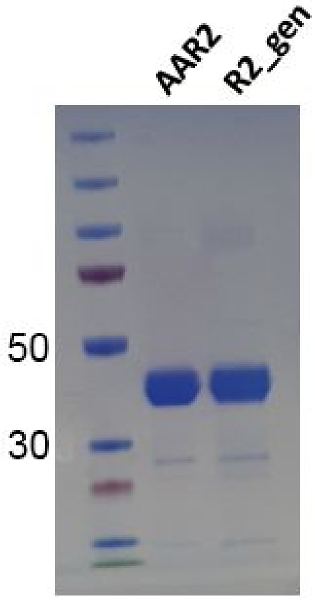

Figure S5. SDS-PAGE analysis of AaR2 and AaR2_genomic proteins following purification by IMAC and SEC. PageRuler Plus Prestained Protein ladder (Thermofischer) was used to estimate the molecular weights of the protein samples. The bands corresponding to 30 and 50 kDa are labelled.

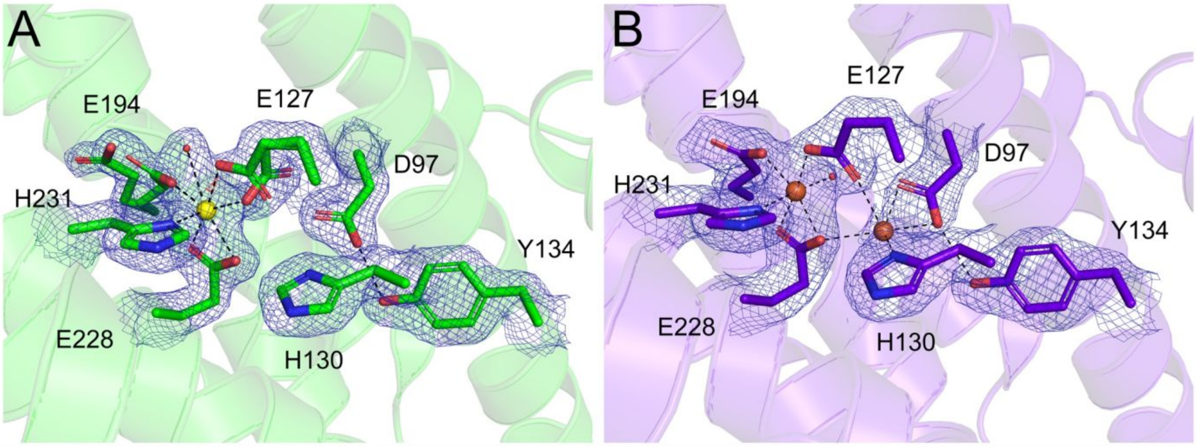

Figure S6. Iron coordination in AaR2 and mature AaR2_genomic. Hydrogen bond network of iron ions within (A) AaR2 and (B) AaR2_genomic. Iron atoms are coloured yellow (AaR2) or orange (AaR2_genomic). Amino acids contributing to metal coordination are depicted as sticks; carbon atoms green (AaR2) or purple (AaR2 genomic), oxygen atoms red and nitrogen atoms blue. The tyrosyl radical residue (Tyr134) in each structure is also shown. Hydrogen bond interactions are displayed as dashed lines. 2*F*o - *F*c electron density maps are contoured at 1.0 σ and *F*o – *F*c electron density maps are contoured at 3.0 σ. Relevant water molecules in each structure are shown as red spheres.

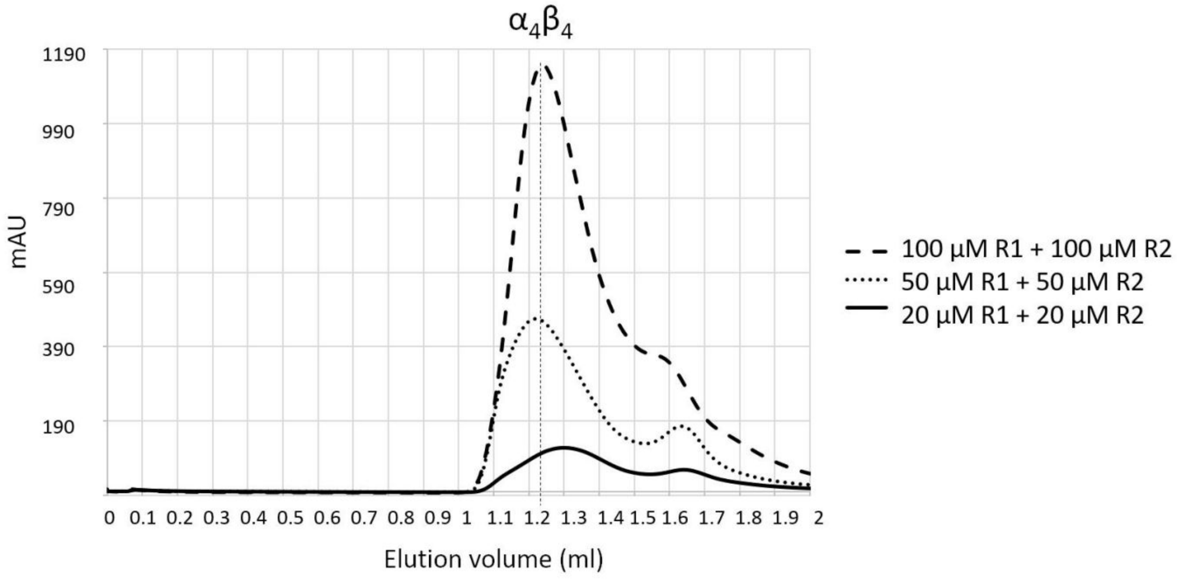

Figure S7. Size exclusion chromatography analysis of AaR1 and AaR2 using different protein concentrations. Experiments were performed in the presence of 5 mM ATP with either 100 µM AaR1 + 100 µM AaR2 (dashed line), 50 µM AaR1 + 50 µM AaR2 (dotted line) or 20 µM AaR1 + 20 µM AaR2 (solid line).
